# Enflicoxib for the long-term management of canine osteoarthritis—External validation of a population pharmacokinetic model in dogs with osteoarthritis

**DOI:** 10.3389/fvets.2025.1645857

**Published:** 2025-09-24

**Authors:** Josep-Maria Cendrós, Marta Salichs, Gregorio Encina, Jose Miguel Vela, Josep Homedes

**Affiliations:** ^1^Biopharmaceutics and Pharmacokinetics Unit, Department of Pharmacy and Pharmaceutical Technology and Physical-Chemistry, Faculty of Pharmacy and Food Sciences, University of Barcelona, Barcelona, Spain; ^2^Ecuphar Veterinarians SLU (Animalcare Group), Barcelona, Spain; ^3^Welab Barcelona, Barcelona Science Park (PCB), Barcelona, Spain

**Keywords:** enflicoxib, NSAID, osteoarthritis, dogs, pharmacokinetics, safety

## Abstract

**Introduction:**

Enflicoxib is a cyclooxygenase-2 (Cox-2) selective non-steroidal anti-inflammatory drug (NSAID) indicated for the treatment of pain and inflammation in canine osteoarthritis (OA) and in soft and orthopedic surgery. Canine OA is a chronic progressive disease that requires long-term therapy.

**Methods:**

The suitability of enflicoxib for its long-term use was assessed by analyzing the concentrations determined in plasma samples from dogs included in a field clinical study using a previously established population pharmacokinetic (popPK) model. One hundred and nine client-owned dogs with OA of different breeds, ages, and weights were enrolled and treated with enflicoxib weekly for 6 months at the recommended dose. Safety was assessed clinically and by repeated blood and urine analysis. Two plasma samples were obtained from 83 dogs that received at least one enflicoxib dose. Concentrations of enflicoxib and its pyrazol metabolite were determined, modeled, and compared with the predictions of the previously established popPK model in healthy Beagle dogs.

**Results:**

Enflicoxib and pyrazol metabolite plasma levels could be adequately predicted by the established popPK model. No covariates other than body weight had any influence on the PK parameters. No over-accumulation of either compound was observed.

**Discussion:**

The established popPK model in healthy Beagle dogs can predict the PK behavior of enflicoxib and its pyrazol metabolite in dogs of any breed with OA. The lack of time-dependent PK provides a PK rationale to support continuous enflicoxib treatment for as long as therapeutically required.

## Introduction

Enflicoxib is a non-steroidal anti-inflammatory drug (NSAID) with notable anti-inflammatory and analgesic activity, belonging to the coxib class of selective cyclooxygenase-2 (Cox-2) inhibitors. Enflicoxib (Daxocox^®^ tablets for dogs, Ecuphar/Animalcare group) is approved for oral treatment of pain and inflammation associated with osteoarthritis (OA) in dogs (1).

The pharmacological properties of enflicoxib in dogs have been described recently. After oral administration, enflicoxib is rapidly metabolized to an active pyrazol metabolite responsible for its extended therapeutic action (2, 3). Its pharmacokinetic (PK) profile has been characterized in Beagle dogs with a mean terminal elimination half-life (t$\frac{1}{2}$) of 20 h for enflicoxib and 17 days for the pyrazol metabolite (4). Based on data from several studies with intensive blood sampling, a mixed-effect population PK (popPK) model was developed to describe the behavior of both compounds in young Beagle dogs (3). The popK model of enflicoxib and its pyrazol metabolite was described by a two-compartment model with first-order absorption (accompanied by a lag time) and elimination for the parent drug, while its pyrazol metabolite followed a three-compartment model with first-order elimination. Only total body weight significantly influenced the PK of both compounds. This popPK model allowed us to conclude that, to rapidly achieve therapeutic plasma concentrations of the active metabolite, enflicoxib should be administered as a first dose of 8 mg/kg, followed by maintenance weekly doses of 4 mg/kg (1). In an overdose study in Beagle dogs, enflicoxib was well-tolerated when administered at 5 times the recommended dose (20 mg/kg) for 3 months or 3 times this dose (12 mg/kg) for 7 months (5), indicating a good safety margin. Periodic blood sampling in this study demonstrated that enflicoxib and its pyrazol metabolite exhibit dose-proportional pharmacokinetics. After 7 months of repeated weekly administrations, pre-dose plasma concentrations (C_min, ss_) remained constant, with no trend to observe any significant over-accumulation (4). Enflicoxib has also been shown to be safe and effective in two field clinical studies for the control of pain and inflammation associated with naturally occurring OA in the target population of dogs, which tend to be elderly large-breed dogs, during 6 weeks of weekly treatment (6, 7).

However, it has been described that some NSAIDs are sensitive to pharmacogenetic and metabolic variability (8–12). Indeed, the variable PK behavior of another long-acting NSAID (mavacoxib), with the existence of subpopulations of slow metabolisers, has prevented its long-term use to avoid the risk of an unpredictable excessive drug exposure (13). This limitation in treatment duration contradicts current recommendations for canine OA management, where continuing NSAID use is advised for as long as necessary or for the lifetime, to maintain functional improvement (14). Therefore, it was judged important to assess the PK variability of enflicoxib and its pyrazol metabolite, not only in laboratory Beagle dogs but also in the target population of dogs with OA, when administered for longer periods of time, with the objective to confirm the suitability of its safe chronic use, according to current recommendations.

This study validates the popPK model in blood samples taken in a third field clinical study conducted to confirm the safety and efficacy of enflicoxib when administered for a period of 6 months in OA patients. The details on the safety and efficacy results of this study are described elsewhere (15).

## Objectives

The main objectives of this pharmacokinetic analysis were to confirm that the pharmacokinetic behavior of enflicoxib and its pyrazole metabolite is comparable between Beagle dogs and dogs with OA and to confirm the absence of time-dependent pharmacokinetics in the target population (dogs with OA).

## Materials and methods

### Clinical study design

A prospective, multisite, blinded, randomized, placebo-controlled, parallel-group field study was performed in accordance with Good Clinical Practice standards (16). The study aimed to confirm the safety and efficacy of enflicoxib treatment of pain and inflammation associated with naturally occurring canine OA in dogs of any breed and sex treated for 6 months. The protocol was approved by the regulatory authorities of both Portugal and Hungary. Written informed consent was obtained from all dog owners prior to enrolment of their dogs. Dogs remained under the care of their owners at home throughout the study.

Full details of the materials and methods of the field clinical study are described in Homedes et al. (15). Briefly, all dogs were client-owned, older than 6 months, and of any breed and sex. On first examination, dogs were required to have clinical signs of OA (pain and lameness) for at least 3 weeks, accompanied by radiographic evidence of OA in at least one joint of the pelvic or thoracic limbs. Exclusion criteria related to non-permitted previous medications or disease-modifying and chondroprotective agents or diets, as well as concomitant diseases, and the concomitant treatment administration restrictions and the applicable withdrawal criteria were as stated in previous efficacy clinical trials with enflicoxib (6, 7).

Before enrolment, all dogs underwent a physical examination, and blood and urine samples were taken. After enrollment, dogs were randomized in a predefined enflicoxib:placebo ratio of 3:1. Enflicoxib was dosed orally at weekly intervals, with an initial dose of 8 mg/kg, followed by once-a-week maintenance doses of 4 mg/kg for a total of 26 weeks. Dogs in the enflicoxib group received Daxocox^®^ tablets (Ecuphar/Animalcare group), while dogs in the placebo group received the same formulation without the active ingredient under the same posology (number of tablets and dosing interval). As food increases absorption (4) and following label indications, enflicoxib or placebo tablets were administered with food or immediately before feeding. No positive control was included due to difficulties in maintaining blinding with products under different posology during the entire follow-up period.

For the clinical efficacy assessment of pain and lameness, a previously defined assessment tool (17) was used by the veterinarian before treatment and at different time points throughout the study. The assessment included different parameters with a final clinical sum score (CSS) ranging from 0 to 12. Efficacy was also assessed by the owners in a blinded condition using the Canine Brief Pain Inventory (CBPI) questionnaire (18, 19). To be eligible for inclusion in the study, a basal CSS≥4 and a PSS and PIS scores ≥2 were required on day 0, prior to treatment.

To assess safety, blood samples for hematology and biochemistry, as well as urine samples, were repeated on days 44 and 189. The following hematological and biochemical parameters were determined: red blood cell (RBC) count, white blood cell (WBC) total and differential count, platelet (thrombocyte) count and estimate, hematocrit, hemoglobin, and reticulocytes. Amylase, albumin, alkaline phosphatase, alanine-aminotransferase (ALT), aspartate-aminotransferase (AST), calcium, cholesterol, creatinine, creatine kinase, globulin, glucose, γ-Glutamyltransferase (γ-GT), magnesium, phosphorus, potassium, sodium, total bilirubin, total protein, and urea.

Urine samples were analyzed for blood/erythrocytes/hemoglobin, glucose, ketone bodies, protein, leukocytes, nitrite, specific weight, pH value, bilirubin, and urobilinogen.

Safety was also assessed by recording any adverse events (AEs) that occurred during the study, regardless of their nature, severity, or whether they were product-related or not.

### Plasma samples

Two blood samples for popPK analysis were collected from all animals enrolled in the study one on day 44(±2 days)/1.5 months, (similar to the last sample taken in the previous clinical studies), after clinical assessment of pain and lameness and prior to treatment administration and another one at the end of the study on day 189/6 months (±2 days), or individual premature study termination day. Day 189 corresponds to the trough concentration after the 27th weekly administration (PK sample taken at the end of the weekly dosing interval). Meanwhile, day 44 corresponds to a PK sample obtained 2 days after the 7th weekly administration. The veterinary practices had the necessary resources and adequate freezing equipment for the long-term storage of plasma samples. Blood samples were collected into potassium-3-ethylenediaminetetraacetic acid (K_3_EDTA) labeled tubes, which were immediately centrifuged at ~2,500*g* for 10 min (at +4 °C whenever possible) to obtain plasma. Once received, plasma was transferred into two labeled propylene tubes (aliquots 1 and 2). Each plasma aliquot was stored frozen at a nominal temperature of −20 (±2) °C until shipment to the Laboratory of analysis Welab Barcelona (Parc Científic de Barcelona, C/Baldiri Reixac 4-12, 08028, Barcelona). The maximum storage time for the samples was 376 days at nominally −20 °C/−80 °C. Considering the long-term stability of enflicoxib and its metabolite in dog plasma samples stored at −20 °C, which spans a minimum of 402 days, the stability of the study samples was guaranteed for the entire duration of the study. Samples were analyzed without information on the treatment allocation (laboratory-blinded study).

### Analytical method

Enflicoxib and pyrazol metabolite plasma levels were measured by a previously fully validated high-performance liquid chromatography (HPLC)-method coupled with tandem mass spectrometry (MS/MS) detection as described in Homedes et al. (4).

The validation of the analytical method demonstrated that it is selective, accurate, precise, reproducible, and linear over the concentration ranges of 5.0–1,000 ng/ml for enflicoxib and 2.5–1,000 ng/ml for the pyrazol metabolite (lower and upper limit of quantification lower and upper limits of quantification (LLOQ–ULOQ), respectively). The method demonstrated an overall precision of < 14.2% and overall accuracy (expressed as a percentage of the nominal value) ranges from 85.0 to 116.1% for both the parent and metabolite. Enflicoxib and pyrazol metabolite were shown to be stable in dog plasma at room temperature for 4 h, at nominally −20 °C for 402 days, and following 4 freeze–thaw cycles.

### Dataset preparation

The PK evaluable population consisted of all animals that received at least one enflicoxib administration. Animals that received a placebo were excluded from the PK dataset. Plasma concentration records of enflicoxib and the pyrazol metabolite, actual sampling times, dosing times, individual actual dose along with individual demographic data (sex, age, breed, and weight), biochemical and hematological parameters were extracted from the clinical database and arranged as a PK dataset using R scripts (R language version 4.2.0 from R Foundation for Statistical Computing).

Independent variables with no recorded actual sampling time were excluded from the popPK analysis unless the nominal time could be considered an adequate representative.

Although there was a small number of missing covariates (< 7% for body weight and < 14% for laboratory measurements), they were handled using the Last Observation Carried Forward (LOCF), or Last Observation Carried Backward (LOCB), as appropriate. In cases where the covariate was not recorded at any time point (only in two dogs for the AST parameter), the mean value was used. Enflicoxib and pyrazol metabolite concentrations were log-transformed to handle the non-normal distribution of residuals. Concentrations below the limit of quantification (BLQ) of both compounds were retained as censored data during the popPK analysis, to implement the M3 approach in NONMEM (NONMEM^®^ version 7.5.0 from ICON Development Solutions). This approach allowed us to simultaneously fit both continuous (measurable drug levels) and categorical data (BLQ), using likelihood estimation only for BLQ data.

### Data analysis and model evaluation

A limited sparse sampling design prevents proper estimation the individual PK parameters when applying the classical non-compartmental analysis. Therefore, the popPK analysis was applied using NONMEM software, considering the population mean parameters along with intersubject and residual variability.

## Modeling strategy

A stepwise approach was applied to estimate individual PK profiles of all dogs included in the field clinical study by means of population methodology.

In the first step, an Exploratory Data Analysis (EDA) was performed, applying graphical techniques to isolate possible patterns and features in the individual PK profiles. Relationship with covariates (sex, weight, age, and blood/biochemical/urine parameters) was also explored.

Secondly, considering that the PK profile of enflicoxib and the pyrazol metabolite could be affected by animal status (healthy vs. osteoarthritic dogs), age, weight and/or influenced by breed, the previously established PK model in healthy Beagle dogs described in Cendrós et al. (3) was tested to assess its applicability to the current study population (dogs of any breed, age, or weight with OA). For this purpose, an external model evaluation was performed applying the visual predictive check (VPC) approach. Individual parameters for the dogs in the field clinical study were estimated using the established PK model in healthy Beagle dogs and compared graphically to the actual observations. Plots of the time course of the field clinical study observations, along with the 90% prediction intervals for the simulated values from the popPK model in healthy Beagle dogs, were visually explored.

As a similar PK behavior between populations was concluded, the same popPK model could be used, and a Bayesian approach was used for the determination of individual enflicoxib and the pyrazol metabolite exposure in osteoarthritic animals.

A maximum a posteriori Bayesian (MAPB) estimation using the *post hoc* subroutine in NONMEM was used to provide the individual PK parameters in osteoarthritic dogs.

MAPB estimation applied likelihood maximization in the form of minimizing the objective function (minus 2 log likelihood), to estimate individual PK parameters of each dog with OA. In this analysis, the MAPB estimation incorporated both *a priori* PK information, that is, the established PK model of enflicoxib and the pyrazol metabolite in healthy Beagle dogs, and a posteriori PK data, which corresponds to PK data from osteoarthritic dogs. The individual PK parameters along with individual predicted PK profiles for each dog treated with enflicoxib were calculated by fitting the animal PK dataset, which includes dosing, sampling times, parent and metabolite levels, and covariate information, to the established population enflicoxib and the pyrazol metabolite PK model in healthy Beagle dogs.

The validity of this Bayesian approach was tested using the following steps:

Diagnostic plots to assess model goodness-of-fit, plotting several graphs, such as: observed concentrations (OBS) vs. population predictions (PRED), OBS vs. individual predictions (IPRED), distribution of conditional weighted residuals (CWRES), etc.; Visual Predictive Check (VPC) to assess graphically whether simulations from the model can reproduce both the central trend and variability in the observed data, when plotted vs. time. For this purpose, at least 500 studies with the same characteristics of the observed data were simulated, and the 95% prediction intervals were calculated for a bin across independent variable by means of PsN (PsN version 5.0.0. from Uppsala Universitet) and Xpose (Xpose version 0.4.13 (R-package) from Uppsala Universitet). Finally, the distribution of simulated drug concentrations was graphically compared with the distribution of the observed dataset; Prediction-corrected Visual Predictive Check (pcVPC), where the dependent variable was subject to prediction correction before the statistics were calculated. The estimation of pcVPC was also implemented in PsN and Xpose; Normalized Prediction Distribution Errors (NPDE): simulation-based diagnostics by means of normalized prediction distribution error (NPDE) were performed. Notably, 1,000 model-predicted concentrations were generated for each observation in the PK dataset, based on simulations that incorporated the dosing regimen and covariates, along with the parameter values (including the interindividual and residual variability) that should be obtained from the original model. The NPDEs, which were estimated using the NPDE add-on package for R, were graphically presented in a total distribution, vs. time and vs. concentration, where no trend is expected, and non-parametric bootstrap: the confidence intervals of the final model PK parameter values were estimated using the non-parametric bootstrap approach implemented in PsN. For the final PK model, at least 250 bootstrap datasets were generated by randomly sampling from the original dataset with replacement. Similar median bootstrap values to the parameter estimates of the original dataset will indicate acceptable parameter precision.

### Established pop PK model in healthy Beagle dogs

Pharmacokinetic analysis was conducted using NONMEM with PsN software as the interface. The established PK model is described in Cendrós et al. (3) using a two-compartment model for the parent compound with first-order elimination and absorption (with lag time), along with a three-compartment model for the pyrazol metabolite with first-order elimination. All terms of variability were reasonably distributed around the zero value with an adequate extent of shrinkage (lower than 30% for all parameters except for interindividual variability on ka and Tlag). The final population PK parameters are shown in Table 1.

**Table 1:** Pharmacokinetic parameter estimates and bootstrap results for the popPK model of enflicoxib and pyrazol metabolite (3).

**Enflicoxib** ^**†**^				**Pyrazol metabolite** ^**‡**^			
**Parameter**	**Units**	**Estimate**	**IIV**	**Parameter**	**Units**	**Estimate**	**IIV**
**Absorption**				**Distribution**			
Relative bioavailability (F)	–	1 FIX	39%	VM/F = θ_4_·(WGT/9.9)^1.0^	–	–	–
Lag Time (Tlag)	hr	0.249	68%	θ_4_	L	6.59	–
Absorption rate (ka)	hr – 1	0.220	101%	VMP/F = θ_9_·(WGT/9.9)^1.0^	–	–	–
Distribution				θ_9_	–	29.3	–
V/F = θ_4_·(WGT/9.9)^1.0^	–	–	94%	VM2/F = θ_13_·(WGT/9.9)^1.0^	–	–	–
θ_4_	L	6.59	–	θ_13_	L	13.4	–
V5/F = θ_11_·(WGT/9.9)^1.0^	–	–	–	CLMP/F = θ_8_·(WGT/9.9)^0.75^	–	–	–
θ_11_	L	27.70	–	θ_8_	L/hr	13.2	–
CL5/F = θ_10_·(WGT/9.9)^0.75^	–	–	–	CLM2/F = θ_12_·(WGT/9.9)^0.75^	–	–	–
θ_10_	L/hr	12.1	–	θ_12_	L/hr	0.131	–
**Elimination**				**Elimination**			
CL2/F = θ_6_·(WGT/9.9)^0.75^	–	–	49%	CLM/F = θ_7_·(WGT/9.9)^0.75^			31%
θ_6_	L/hr	0.520	–	θ_7_	L/hr	0.111	–
CL1/F = θ_5_·(WGT/9.9)^0.75^	–	–	–	–	–	–	–
θ_5_	L/hr	0.193	–	–	–	–	–
Residual variability	%	34%	–	Residual variability	%	20%	–

^†^For parent enflicoxib: relative bioavailability (F); absorption rate (Ka); lag time (Tlag); apparent volume of distribution in the central compartment (V/F); apparent volume of distribution in the peripheral compartment (V5/F); apparent clearance to other elimination pathways (CL1/F); apparent clearance of irreversible biotransformation to pyrazol metabolite (CL2/F) and apparent distribution clearance between central and peripheral compartment (CL5/F).

^‡^For the pyrazol metabolite: apparent volume of distribution in the central compartment (VM/F); apparent volume of distribution in the shallow peripheral compartment (VMP/F); apparent volume of distribution in the deep peripheral compartment (VM2/F); apparent clearance (CLM/F); apparent distribution clearance between central and shallow peripheral compartments (CLMP/F), and apparent distribution clearance between central and deep peripheral compartments (CLM2/F).

In this popPK model, the interindividual variability (IIV) associated with the PK parameters was modeled assuming a log-normal distribution as follows:


$$\begin{array}{l} P_{i}=P_{POP}\cdot e^{\eta_{i}} \end{array}$$

where *P*_i_ represents the value of the PK parameter for individual *i*; *P*_POP_ is the typical population estimate for the PK parameter, and h_i_ is a random variable that accounts for the deviation between individual value (*P*_i_) and typical population value (*P*_POP_). The set of η_i_ is assumed to be independent and symmetrically distributed around 0 with variance ω^2^. The magnitude of IIV is expressed as the coefficient of variation (% CV).

Residual variability, which accounts for random unexplained discrepancies between observed and individual predicted concentrations, was modeled using a log error model as shown below:


$$\begin{array}{l} \ln\;C_{ij}=\ln\;C_{pred,ij}+\varepsilon_{ij} \end{array}$$

where *C*_*ij*_ and *C*_*pred, ij*_ represent the *j-*th observed and model-predicted enflicoxib or the pyrazol metabolite concentrations, respectively, for individual *i*. Also, *e*_*ij*_ denotes the additive residual random error for individual *i* and observation *j*. The ε random effects are assumed to be independent and symmetrically distributed with a mean of zero and a variance equal to *s*^2^. The magnitude of residual variability, which was expressed as % CV, can be attributed to, but not limited to, drug assay variability, protocol deviations (sampling, dosing, etc.), intraindividual variability, and model misspecification.

The established pop PK model in healthy Beagle dogs includes the total body weight as a covariate in clearances and volume of distributions using the following allometric equation:


$$\begin{array}{l} P_{POP}=\;\theta_{1}\cdot {\left(\frac{\text{WGT}}{\text{median}\left(\text{WGT}\right)}\right)}^{\theta_{2}} \end{array}$$

where *P*_POP_ represents the typical value of a PK parameter, WGT represents the individual weight; median (WGT) is the median total body weight; and θ_1_ and θ_2_ correspond to the allometric coefficient and allometric exponent, respectively.

A covariate was included in the model if it reduced the objective function by at least 3.84 points (*p* < 0.05, forward selection) or 7.88 points (*p* < 0.01, backward elimination).

## Results

### Study population

One hundred and nine dogs with clinical and radiographic signs of OA were enrolled in the field clinical study. The 26 dogs receiving a placebo were not included in the database. Only the 83 dogs [48 males (57.8%) and 35 females (42.2%)] from the active-treatment group that received at least one dose of enflicoxib were included in the PK dataset. Forty-two dogs (50.6%) were purebred and 41 mixed-bred (49.4%), with more than 25 other breeds represented (the highest frequency being the Labrador Retriever and German Shepherd). Seventeen dogs received a variety of medications that were administered concurrently with enflicoxib. The basal overall mean ± standard deviation (SD) age of the dogs in this group was 8.7 ± 3 years with a range of 2–16 years. The mean ± SD body weight of the dogs was 27.0 ± 15 kg, with a range of 4.9–64.9 kg.

Fifty-seven dogs received the 27 oral weekly drug administrations scheduled by protocol. Four dogs received an additional dose by mistake, and 22 dogs missed at least one scheduled dose (ranging from 1 to 26 missing doses). Total dose administered was modified during the study in only three dogs to adjust to the increase in body weight.

### Clinical outcomes

In this field clinical study, the efficacy of enflicoxib was similar to what has been reported in previous field clinical studies of shorter duration (6, 7). As efficacy was not used for any PK adjustment, no further detail is provided here.

Regarding safety, the results obtained for the hematological and biochemical parameters were generally within the reference ranges of the laboratory of analyses. No statistically significant differences were observed in any parameter, except for a slight increase in average urea values on Day 189, although within the laboratory reference range. Likewise, the incidence and type of adverse events were as described in previous enflicoxib studies of shorter duration (6, 7) and similar to those of other NSAIDs, with no tendency to increase over time. A thorough description of the safety results can be found in Homedes et al. (15). See details in Supplementary Data.

### Plasma samples

Not all animals strictly accomplished the PK sampling times scheduled in the study protocol. Eight dogs were withdrawn from the study before the first sampling time on day 44; therefore, they had no samples for the PK analysis. Likewise, 8 dogs were withdrawn before day 189 and had only one plasma sample taken on day 44. Two more dogs were also withdrawn before day 189, but they had the second plasma sample taken on day 93, which corresponds to the premature study termination day. All other dogs (65) had the two samples taken, one on day 44 and one on day 189. Sampling days ranged from 42 to 49 days for the first sampling time and 182–196 days for the second sampling time.

### Exploratory data analysis

#### Enflicoxib and pyrazol metabolite plasma levels

Table 2 shows enflicoxib and pyrazol metabolite mean plasma levels obtained on days 44 and 189. Drug levels on day 93 are also tabulated, which corresponds to the two dogs that early terminated the study. The percentage of samples below LOQ is shown, and the percentage of samples below 2 times the highest LOQ (< 10 ng/ml) is also included as a reference to detect the unexpected low concentrations. The higher percentage of < LOQ of enflicoxib on day 189 compared to day 44 was due to the different sampling times related to the last product administration (day 189 PK sample was taken at the end of the weekly dosing interval, meanwhile day 44 corresponds to a PK sample obtained 2 days after the seventh weekly administration).

**Table 2:** Enflicoxib and pyrazol metabolite plasma levels on days 44, 93, and 189.

**Compound**	**Theoretical visit day**	**Mean (ng/mL)**	**SD (ng/mL)**	**% CV**	**Min (ng/mL)**	**Max (ng/mL)**	***N***	**Probability (%) concentration < LOQ**	**Probability (%) concentration < 10 ng/mL**
Enflicoxib	44	4,917	462.5	94.1	5.0	1,846.5	75	9.3	13.3
	93	296.8	83.6	28.2	237.7	356.0	2	0.0	0.0
	189	262.5	393.5	149.9	5.0	1,846.3	65	27.7	27.7
Pyrazol metabolite	44	2,105.6	1,071.7	50.9	2.5	4,728.5	75	4.0	4.0
	93	3,928.8	1,556.0	39.6	2,828.5	5,029.0	2	0.0	0.0
	189	2,317.4	1,591.8	68.7	2.5	7,541.8	65	1.5	4.6

**Figure 1:**
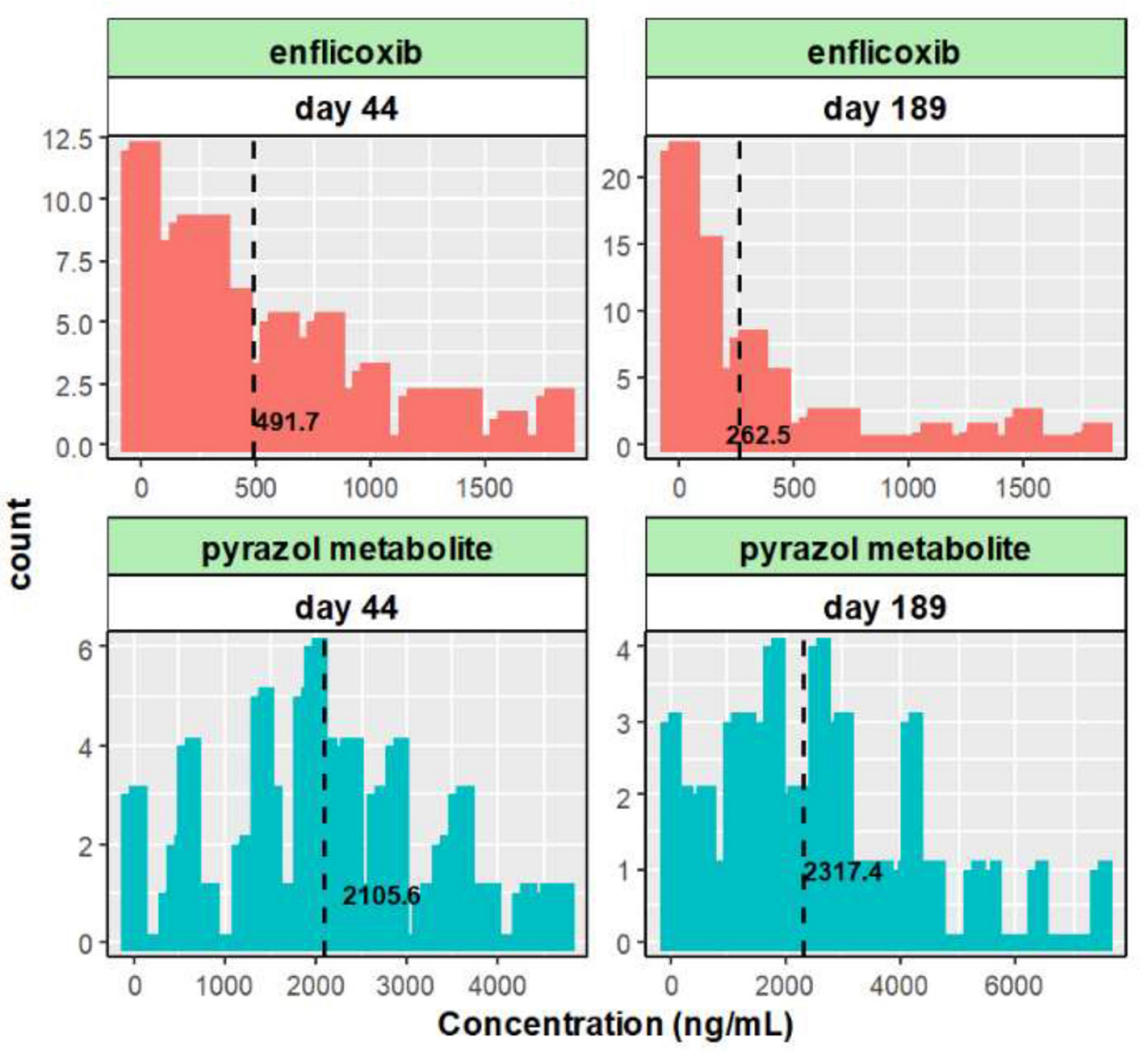
Histogram of the enflicoxib and the pyrazol metabolite plasma levels obtained on day 44 and 189. Dashed lines represent average values.

The distribution of enflicoxib and pyrazol metabolite plasma levels on day 44 and day 189 is depicted in Figure 1. While right-skewed distributions were observed for enflicoxib on days 44 and 189, close to normal distributions were observed for the pyrazol metabolite on both days. A few high plasma levels were observed on day 189 for both compounds, as 5 animals presented enflicoxib plasma levels higher than 1,000 ng/mL, and 4 reached pyrazol metabolite levels above 5,000 ng/mL. These figures also show that the number of samples with low drug levels was much lower for the pyrazol metabolite than for enflicoxib. The differences in the distribution of both compounds reflect the distinct terminal half-life of each compound: the parent (1.4 days) is cleared faster than the pyrazol metabolite (13.8 days).

#### Relationship between plasma levels and covariates

The drug plasma level relationships with the main continuous covariates (demographic, hematological and biochemical characteristics) and four categorical covariates (sex, breed, affected joint and vomiting), were initially considered. However, although breed-related differences can influence drug PK and concurrent medications could interact with enflicoxib, the use of breed or concomitant medications as categorical covariate was discarded due to a large variety of cases.

On the other hand, emesis could influence the PK profiles of any drug if it occurs within 12 h after dosing when absorption may not be complete. In this study three dogs experienced at least one episode of vomiting. However, these episodes were registered at least 1 day after product administration without pharmacokinetic implications except for one dog where vomiting was observed the same day of the 14th administration on day 91. After this episode, the dog received 13 additional weekly doses, up to the end of the study on day 189, when a plasma sample for PK was obtained, with no further episodes of vomiting. Therefore, considering that the exposure achieved on day 189 is unlikely to be affected by the vomiting episode on day 91, it was decided not to exclude this dog from the PK analysis.

No relationship or very weak correlations were observed for all covariates, except for body weight (Supplementary Figure S1 depicts the correlation between the most relevant covariates and plasma levels of enflicoxib and the pyrazol metabolite on days 44 and 189). A clear trend of increased enflicoxib and pyrazol metabolite plasma levels with increasing body weight was observed on days 44 and 189, being more evident for the pyrazol metabolite. For the remaining covariates, no clear trends were observed.

#### PK comparison between healthy Beagle dogs and dogs with OA

Considering that animal status could influence the PK of both enflicoxib and the pyrazol metabolite, the predictions using the previously established PK model based on young healthy Beagle dogs were compared to the actual plasma levels obtained in this study in aged osteoarthritic dogs of any weight and age.

**Figure 2:**
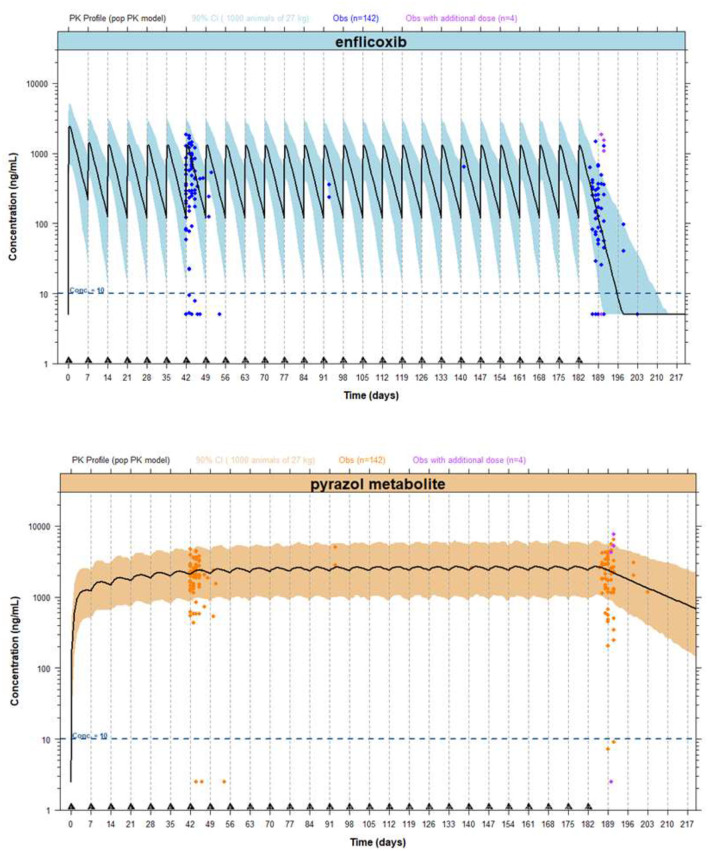
External evaluation plots, including PK profiles of enflicoxib and the pyrazol metabolite, after administration of enflicoxib at a first dose of 8 mg/kg followed by a weekly maintenance dose of 4 mg/kg for 26 weeks. Typical predicted PK profile of enflicoxib and the pyrazol metabolite (solid line) along with 90% prediction intervals (colored area) obtained by the established popPK model in healthy Beagle dogs compared with drug observations in dogs of any breed with OA (symbol).

An external evaluation, applying the visual predictive check (VPC) approach, was implemented for each compound using the characteristics of this study on the PK model: mean body weight of 27 kg, first dose of 8 mg/kg and maintenance weekly doses of 4 mg/kg administered for 6 months (see Figure 2). For simulation purposes, the mean of the actual recorded doses was used in place of the theoretical doses: an initial dose of 10.4 mg/kg and a maintenance dose of 5.2 mg/kg. Although some PK discrepancies were observed between simulations and observations, it appears that the PK model in healthy Beagle dogs can accurately reproduce the PK behavior of enflicoxib and the pyrazol metabolite in dogs with OA. The observed data for both compounds are randomly distributed throughout the simulated mean PK profile, and the majority of the observations lie within simulated bounds. It should be noted that using actual rather than fixed exact sampling times can lead to higher dispersion of observed plasma levels over time for both compounds.

The mean simulated concentrations of enflicoxib were somewhat higher than observed on day 44 (765 and 492 ng/mL, respectively) and somewhat lower than observed on day 189 (118 and 263 ng/mL, respectively); however, the majority of the individual observations fell within the 90% confidence interval. The lower observed concentrations of enflicoxib are likely due to the greater variability inherent in clinical field studies in dogs.

Due to the nature of a long-field clinical study compared to laboratory studies in which the PK model is based, it was expected that the observed variability on enflicoxib and the pyrazole metabolite plasma levels would be somewhat higher than that predicted by the PK model.

In fact, 5 PK samples were well outside the upper 90% confidence interval on day 189. However, three out of these 5 PK samples correspond to dogs that received, by mistake, an additional administration on day 189 that was not included in the simulations.

On the other hand, 13% (10/75) of PK samples on day 44 and 28% (18/65) of PK samples on day 189 provided unexpectedly low enflicoxib plasma levels (below 10 ng/mL). Percentages of unexpectedly low enflicoxib levels (below 10 ng/mL) predicted by the PK model, compared with those observed, were somewhat lower on day 44 and similar on day 189.

The PK model simulates that none of the treated dogs will present pyrazol metabolite levels below 10 ng/mL; however, 3 dogs showed levels below 10 ng/mL on day 44 and 3 different dogs on day 189, representing a very low percentage of samples for each sampling time (only 4 and 5%, respectively). Considering that no missing doses were reported for these dogs, there is no PK reason to justify these low pyrazol metabolite levels. However, as previously commented, it was expected that the observed variability in the pyrazol metabolite plasma levels would be higher than that predicted by the model.

In addition, a group of animals presented pyrazol metabolite levels above 10 ng/mL but below the lower simulated confidence interval (~700 ng/mL) on either day 44 (*n* = 9) and/or day 189 (*n* = 9). Only four animals presented these low levels on both days (44 and 189); meanwhile, the remaining dogs presented these values on day 44 or on day 189.

##### Bayesian approach to predict the individual drug exposure of enflicoxib and the pyrazol metabolite for each osteoarthritic dog included in the treated group

The maximum a posteriori Bayesian (MAPB) estimation was applied to provide the most likely individual PK profile for each dog with OA, using the “POSTHOC” subroutine and $ESTIMATION command set to MAXEVAL=0 in NONMEM.

**Figure 3:**
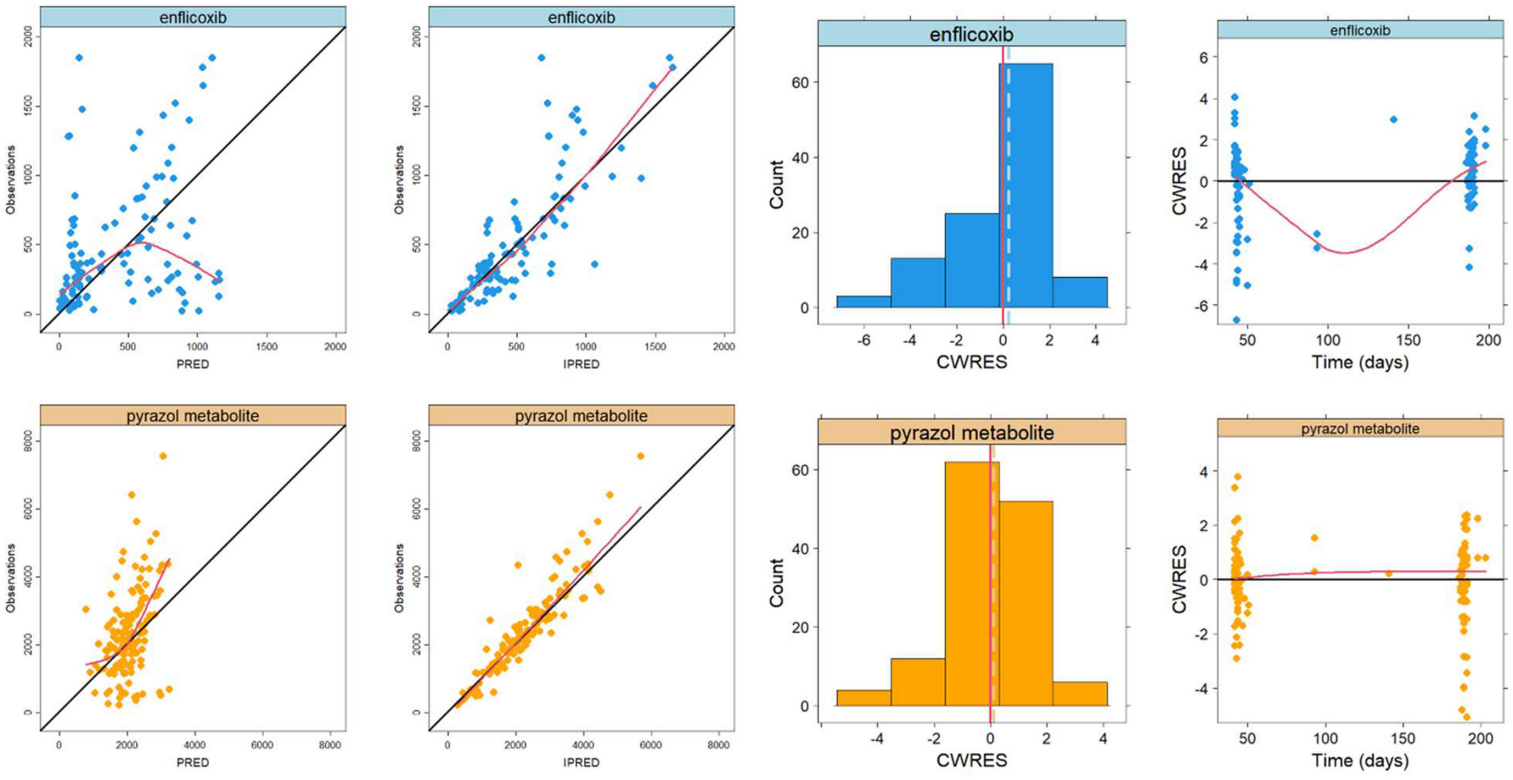
Basic goodness-of-fit plots for Bayesian estimation of enflicoxib **(top panels)** and the pyrazol metabolite **(bottom panels)** in dogs of any breed with OA. Plotted lines correspond to the line of identity (black) and the non-parametric smooth curve (red).

The basic goodness-of-fit plots for Bayesian estimation of enflicoxib and pyrazol metabolite plasma levels in dogs suffering from OA are depicted in Figure 3.

Overall, the established PK model of enflicoxib and the pyrazol metabolite in healthy Beagle dogs adequately described the drug data from dogs with OA. These plots revealed good agreement between the observed and population- and individual-predicted values (PRED and IPRED, respectively), showing that the majority of the data points for enflicoxib and the pyrazol metabolite were symmetrically distributed around the line of identity. In addition, the normal distribution with a mean close to zero for the conditional weighted residuals (CWRES), along with the lack of noticeable trends in the CWRES vs. time for enflicoxib and the pyrazol metabolite, indicates the appropriateness of the residual model and the adequacy of the structural PK model, respectively.

Results of goodness of fit plots confirmed that the PK behavior of enflicoxib and the pyrazol metabolite in dogs suffering from OA is similar to that of healthy Beagle dogs, and thus, confirmed the adequacy to apply the Bayesian approach to estimate individual PK parameters of dogs included in the field clinical study. The data are randomly distributed around the reference lines, indicating no meaningful trends. Apparent deviations in the spline at the extremes are likely due to sparse data at those time points, rather than reflecting actual patterns.

Individual PK parameters and individual PK profiles of enflicoxib and the pyrazol metabolite were estimated for each dog. These results were obtained by fitting the PK data to the established popPK model in healthy Beagle dogs, taking into account the dosage history and covariates (in this case, only the total body weight) by using Bayesian approach.

Supplementary Figure S2 shows the individual predicted PK profiles of enflicoxib and the pyrazol metabolite for each dog treated with enflicoxib. Twenty-two dogs presented an incomplete PK profile because they did not receive all scheduled doses, and 4 dogs were treated with an unscheduled additional dose on day 182. In addition, the population approach also allowed for the prediction of the typical PK profile in dogs without any plasma levels but with dose and body weight information.

There were 10 dogs that presented a lower pyrazol metabolite level than the 5% confidence interval simulated by the PK model. This finding could be partially explained because a fixed maintenance dose was used to perform the simulations (5.2 mg/kg), and four animals were treated with a lower dose level. For the remaining dogs, these low pyrazol metabolite values can be attributed to the interindividual variability. These dogs, however, achieved adequate clinical efficacy in the study and were considered responders under both veterinarian and owner criteria.

The fact that, as predicted, similar mean levels of pyrazol metabolite were achieved on days 44 and 189 indicates that the PK steady-state was attained, and thus no time-dependency was observed after 6 months of treatment, suggesting that progression of OA has no impact on pharmacokinetics.

Finally, the Bayesian approach enabled the estimation of the main individual PK parameters of enflicoxib and the pyrazol metabolite. Using these individual PK parameters, predicted profiles for each animal were estimated, allowing the calculation of the derived exposure parameters, such as *C*_max_, *C*_min_, AUC_τ_, and *T*_1/2_ in all treated dogs. The distribution and the mean estimates of enflicoxib and the pyrazol metabolite PK parameters at selected time points are depicted in Supplementary Figures S3, S4, respectively.

Regarding enflicoxib PK parameters, the *C*_max_ presented a similar value (≈1,173 ng/mL) and similar normal distribution during all weeks of treatment. *C*_min_ and AUC_τ_ showed a similar pattern to *C*_max_, with a similar mean value across weeks (~185 ng/mL for *C*_min_ and 3,810 ng/mL·d for AUC_τ_), but with an increase of interindividual variability. This increase in variability was higher for *C*_min_ than for AUC_τ_, with a higher right-skewed distribution for *C*_min_. These results indicate that the steady-state of enflicoxib is reached with 4 weeks of weekly treatment, and it is maintained throughout the study.

For the pyrazol metabolite PK, a similar pattern was observed for *C*_max_, *C*_min_, and AUC_τ_, maintaining similar values from weeks 12–26 (~2,490 ng/mL, 2,243 ng/mL, and 16,742 ng/ml·day, respectively) with a normal distribution. These findings indicate that the PK steady-state of the pyrazol metabolite is attained after 10–12 weeks of weekly treatment, and it is maintained during at least 26 weeks.

Three dogs (3.6%) showed a somewhat longer terminal half-life for both enflicoxib and the pyrazol metabolite. However, the plasma concentrations of these animals were within the variability predicted by the popPK model for both compounds. Therefore, there is no evidence of time-dependent pharmacokinetics or over-accumulation for either compound.

**Figure 4:**
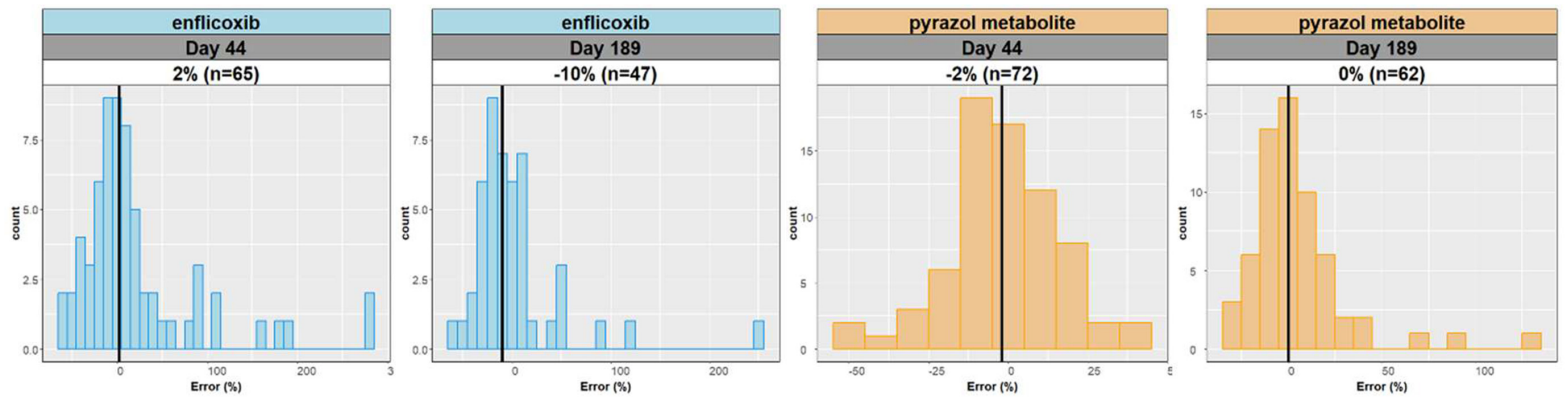
Distribution of relative errors (%) between individual predictions and observations for enflicoxib and the pyrazol metabolite on days 44 and 189.

Confirming similarity between predictions and observations, the median error between individual predictions and observations for enflicoxib and the pyrazol metabolite is close to zero with a normal distribution on days 44 and 189 (see Figure 4), being more consistent for the pyrazol metabolite.

#### Bayesian PK model evaluation

**Figure 5:**
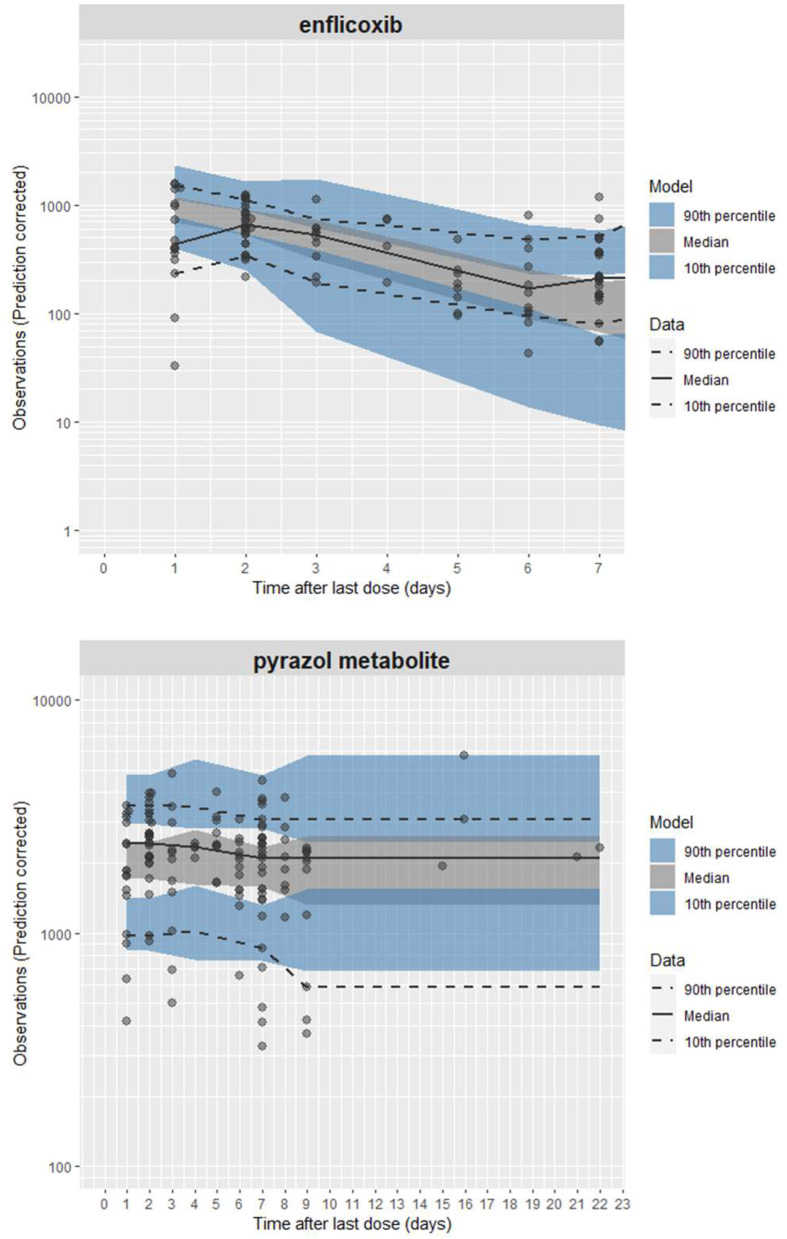
Prediction-corrected visual predictive check (pcVPC) of the Bayesian PK model for enflicoxib and the pyrazol metabolite vs. time after last dose (TALD). The black solid line represents the median of the raw data, the black dotted lines are the 5th and 95th percentiles of the raw data, and the shaded areas are the 90% prediction intervals of the 5th, 50th, and 95th percentiles of the 1,000 simulations based on the established. PK model on healthy Beagles. The open gray circles represent the predicted corrected observations in dogs of any breed with OA.

To obtain a global overview of the validity of the Bayesian estimation, the prediction-corrected Visual Predictive Check (pcVPC) for enflicoxib and the pyrazol metabolite was computed and is displayed in Figure 5. The red and blue bands represent the 95% prediction intervals for the median, 2.5th and 97.5 percentiles of simulated data. In addition, the prediction-corrected concentrations (blue circles) and the median (solid red line), along with 2.5th and 97.5th percentiles of prediction-corrected observations (dotted red lines), were also superimposed. The left panel shows that, although the majority of the observed enflicoxib levels are inside the predicted variability, there was some degree of inconsistency between observations (black lines) and predictions (shaded areas) for enflicoxib, where limited PK data are available due to the higher percentage of < LOQ values, but without observing a clear trend. On the other hand, the right panel plot illustrates that the observed pyrazol metabolite data fall largely within the simulated range, with the 5th, 50th, and 95th percentiles of the observed data in close agreement with the corresponding prediction intervals of the simulations. Overall, these results indicate that the PK model can adequately predict the PK profiles of enflicoxib and the pyrazol metabolite in dogs with OA.

**Figure 6:**
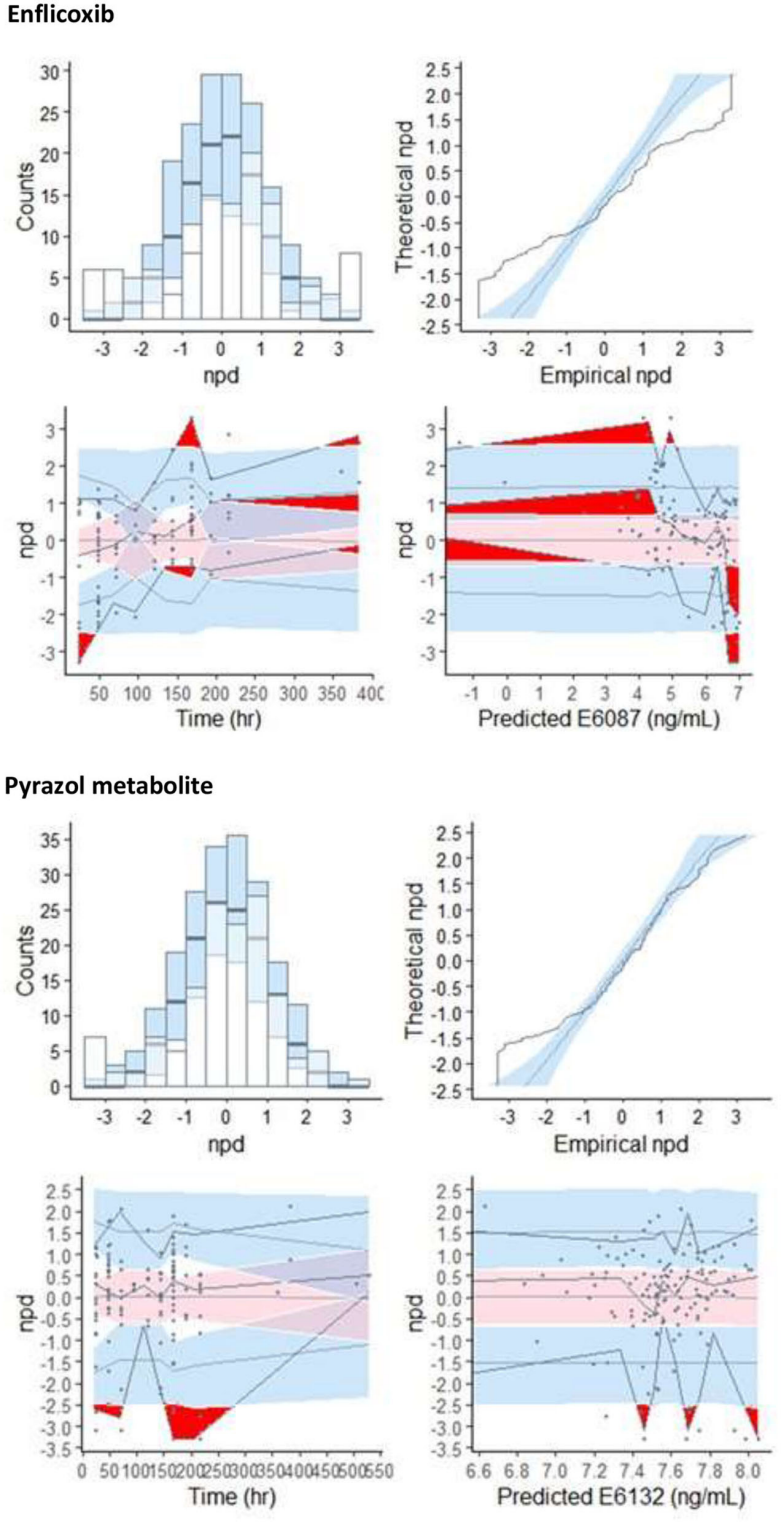
NPDE model evaluation plots for enflicoxib and the pyrazol metabolite: Histogram of NPDE with the density of the standard normal distribution overlaid **(top left panel)**, Quantile-Quantile plot of NPDE vs. the expected standard normalized distribution **(top right panel)**. Scatterplot of NPDE vs. observed time after last dose **(bottom left panel)** and NPDE vs. predicted concentration **(bottom right panel)**.

The results of the normalized prediction distribution errors (NPDE) analysis for enflicoxib and the pyrazol metabolite are depicted in Figure 6. Overall, the NPDE qualification results showed some trends in the enflicoxib predictions but limited biases for the pyrazol metabolite, suggesting an acceptable match between simulations and observations. Adequate distribution of NPDE was observed. The discrepancy between predicted and observed values for enflicoxib and the pyrazol metabolite can be assumed to be normally distributed (top panels). The NPDE vs. time and NPDE vs. predicted levels in log scale (bottom panels) presented some biases for enflicoxib, where limited PK data are available; meanwhile, the pyrazol metabolite plots did not indicate any major prediction deficiencies.

**Figure 7:**
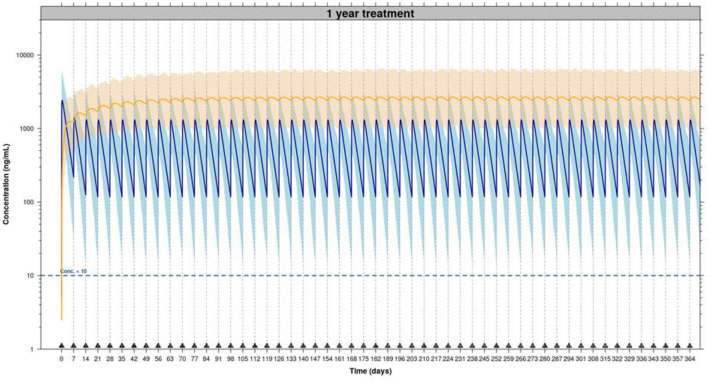
Simulated PK profiles of enflicoxib and the pyrazol metabolite after administration of enflicoxib at a first dose of 8 mg/kg followed by a weekly maintenance dose of 4 mg/kg for 1 year (53 weeks). Typical predicted PK profile of enflicoxib and the pyrazol metabolite (solid line) along with 90% prediction intervals (colored area) obtained by established popPK model.

The results obtained from these evaluation techniques confirm the adequacy of the established PK model in healthy Beagle dogs to describe the PK behavior of enflicoxib and the pyrazol metabolite in dogs with OA after repeated oral weekly administration of enflicoxib. This population PK model is also suitable to simulate the PK profile of enflicoxib and pyrazol metabolite after a long-term weekly administration of 4 mg/kg in dogs with OA (see Figure 7 for a 1-year simulation). No time-dependent PK changes are observed after simulating long-term administration, as once the pharmacokinetic steady state is reached for both compounds (4 weeks for enflicoxib and 12 weeks for pyrazol metabolite), the simulated PK profiles remain constant. No over-accumulation of enflicoxib and pyrazol metabolite is expected.

## Discussion

This population PK analysis for enflicoxib and its pyrazole metabolite was undertaken because the preclinical laboratory studies [pharmacokinetic, pharmacodynamic, and safety (3–5), respectively] had been performed in young healthy Beagle dogs, which have quite different characteristics from the intended target population of dogs of different breeds suffering from OA. Indeed, it has been demonstrated that NSAIDs such as celecoxib, mavacoxib, and cimicoxib show high within and/or between-breed variability in the dog (9, 11, 12, 20, 21). In the case of mavacoxib, high between-subject variability and associations between apparent clearance (Cl/F) and age, breed, and notably body weight were observed (11). Additionally, a higher exposure of robenacoxib was observed in a population of mixed-breed osteoarthritic dogs compared to healthy young Beagle dogs, which was attributed to a cytochrome P450 inhibition due to chronic inflammation (22).

Because of the potential impact any variability in plasma levels could have on efficacy and safety, it was considered important to characterize the pharmacokinetic variability of enflicoxib and its pyrazol metabolite in the target population (frequently large-breed elderly dogs) and to document whether any covariates could play a significant role in this variability. Also, the latest trends recommending continuous pain control in dogs affected with OA, particularly the need for sustained long-term treatment with NSAIDs (14), recommend assuring the adequacy of enflicoxib and its weekly posology under continuous treatments.

Using classical statistical analysis, an intense blood sampling is required for a proper pharmacokinetic evaluation. However, in field studies performed with the target population of client-owned dogs with OA, it is not ethically feasible. Therefore, the use of a popPK approach allowed the characterization of enflicoxib and its metabolite pharmacokinetics in osteoarthritic patients included in a field clinical study with limited blood sampling.

The increase of interindividual variability (measured as CV%), which was observed for both compounds (enflicoxib and the pyrazol metabolite) as treatment progresses from day 44 to day 189, can be partially explained by the use of theoretical rather than actual sampling times and by the expected increase of uncertainty as the treatment period increases.

For enflicoxib, the mean drug levels observed on day 44 were higher than those on day 189 (491.7 and 262.5 ng/mL, respectively). This finding could be attributed to the fact that day 189 corresponds to the trough concentration after the 27th weekly administration (PK sample at the end of dosing interval [τ = 7 days]) while day 44 drug levels correspond to a sample obtained only 2 days after the 7th weekly administration. Another reason can be attributed to the higher number of PK samples below the limit of quantification observed on day 189 compared to day 44 (27.7 and 9.3%, respectively), which is consistent with the half-life of parent enflicoxib (4) and the time elapsed from the last administration to sampling.

For the pyrazol metabolite, the mean plasma concentrations tend to increase slightly from day 44 to day 189 (2,105.6 and 2,317.4 ng/mL, respectively), which is compatible with the fact that steady state has not been yet reached on day 44 due to the long terminal half-life (*t*_1/2_ = 13.8 days) for the pyrazol metabolite (4).

A small percentage of dogs presented concentrations above or below the expected range. Lower values may be a consequence of the implicit higher variability of the real dose administered, mode and time of administration, and sampling time points within a long-term field clinical study with privately owned dogs compared to an intensively monitored laboratory trial. In majority of these cases, the efficacy was satisfactory, so they have to be considered within normal variability. Some of the highest values have similar explanations, and from the safety point of view, even the highest concentrations are below those found in the Target Animal Safety study (5), where Beagle dogs treated with 5 times the maximum recommended dose (20 mg/kg) weekly for 3 months did not show any clinical signs nor biochemical alterations and achieved pyrazol metabolite plasma levels >6,000 ng/mL. Moreover, these concentrations were not associated with any increase in AEs, and the incidence of AEs decreased with time, despite the concentrations of the active pyrazol metabolite tended to slightly increase.

The lack of correlation between plasma concentrations and any of the safety variables confirms the safety of long-term enflicoxib in the target population. In agreement with other NSAIDS of the same class, no effect has been seen for age or sex (23). Liver or kidney function was not affected by the treatment; however, all dogs included in the field clinical study had normal biochemical parameters as an inclusion criterion. Therefore, no inference can be made on the PK behavior of enflicoxib in dogs with impaired liver or kidney function.

A mixed-effect pharmacokinetic model was developed for enflicoxib pharmacokinetics in experimental Beagle dogs (3), and in this study, it was proven to be applicable to dogs with naturally occurring OA. Unlike the case of mavacoxib and robenacoxib (20, 23), no differences were found in the PK behavior of healthy young Beagle dogs compared to the older osteoarthritic dogs, other than those related to weight. In fact, the established popPK model, which has been validated in healthy Beagle dogs, already incorporates allometric exponents in clearances and volumes of distribution (0.75 and 1.00, respectively) to estimate PK parameters for any weight (3). As it was already seen in Beagle dogs, plasma concentrations of the majority of the animals in this study were within the variability predicted by the popPK model for both compounds, and no evidence of time-dependent pharmacokinetics or over-accumulation for either compound was identified, even when this prediction is extended to 1 year of continuous weekly administration.

The popPK model selected enabled the effective analysis and comparison of the sparse data obtained in dogs with OA from a field clinical study with the richer dataset of the smaller experimental Beagle dog population. The reliability and consistency of the predictions from the popPK model reassure the safe use of enflicoxib in the long-term management of pain and inflammation related to canine OA. Obtaining real-world data from dogs with OA treated over a 6-month period would be valuable for fully validating long-term PK predictions.

## Conclusion

The established popPK model in healthy Beagle dogs can be used to project the PK behavior of enflicoxib and the pyrazol metabolite in dogs of any breed with OA.

The lack of time-dependent PK or unexpected over-accumulation for enflicoxib and the pyrazol metabolite in dogs with OA provides a PK rationale to support the use of enflicoxib for as long as therapeutically required.

## Data Availability

The datasets used and/or analyzed during the current study are available from the corresponding author on reasonable request. Requests to access the datasets should be directed to jhomedes@ecuphar.es.
