# Supplementary material for: Enflicoxib for the long-term management of canine osteoarthritis—External validation of a population pharmacokinetic model in dogs with osteoarthritis
Source: Front Vet Sci. 2025 Sep 24;12:1645857. doi: 10.3389/fvets.2025.1645857 (PMC12505664; doi:10.3389/fvets.2025.1645857)
Supplement: Supplementary file 1 [file Data_Sheet_1.pdf]

Supplementary data

Haematological and biochemical parameters and digestive adverse events as described in:

Homedes J, Ocak M, Riedle S, Salichs M. A blinded, randomized and controlled multicenter field study investigating the safety and efficacy of long-term use of enflicoxib in the treatment of naturally occurring osteoarthritis in client-owned dogs. Front Vet Sci. 2024 Feb 23;11:1349901.

Selected haematology parameters mean values for dogs treated with enflicoxib orally once a week for 6 months (SD)

Treatment group		Enflicoxib	*n*	Placebo	*n*	P †
Red blood cell count	Basal	7.2 (1.0)	*81*	7.3 (0.7)	*26*	ns
($x10^{12}$/L) (RBC)	Day 44 (1,5 months)	7.2 (1.1)	*75*	7.6 (0.8)	*26*	ns
	Day 189 (6 months)	7.0 (0.9)	*64*	7.3 (1.1)	*24*	ns
Reticulocytes	Basal	73.0 (42)	*42*	74.5 (31)	*12*	ns
(x10^3/ul) (Retic)	Day 44 (1,5 months)	75.2 (37)	*39*	73.4 (32)	*14*	ns
	Day 189 (6 months)	59 (30)	*28*	77.6 (25)	*13*	ns
Hemoglobin (g/L)	Basal	164 (20)	*81*	166 (14)	*26*	ns
(Hb)	Day 44 (1,5 months)	163 (20)	*75*	172 (14)	*26*	ns
	Day 189 (6 months)	159 (18)	*64*	164 (23)	*24*	ns
Hematocrit (%)	Basal	50.1 (7.7)	*81*	51.6 (5.7)	*26*	ns
(Hct)	Day 44 (1,5 months)	49.7 (7.5)	*75*	52.9 (5.3)	*26*	ns
	Day 189 (6 months)	49.5 (6.6)	*64*	51.7 (7.8)	*24*	ns
Platelets ($x10^9$/L)	Basal	292 (123)	*81*	320 (157)	*26*	ns
(Hct)	Day 44 (1,5 months)	295 (131)	*75*	356 (178)	*26*	ns
	Day 189 (6 months)	274 (93)	*64*	292 (170)	*24*	ns

Selected blood chemistry parameters mean values for dogs treated with enflicoxib orally once a week for 6 months (SD).

Treatment group		Enflicoxib	*n*	Placebo	*n*	P[†]
Alkaline phosphatase (U/L)	Basal	107 (96)	*81*	97 (65)	*26*	ns
(ALP)	Day 44 (1,5 months)	95 (73)	*75*	111 (88)	*26*	ns
	Day 189 (6 months)	84 (52)	*64*	80 (47)	*24*	ns
Alanine aminotransferase (U/L)	Basal	52 (31)	*83*	52 (44)	*27*	ns
(ALT)	Day 44 (1,5 months)	48 (31)	*81*	44 (30)	*28*	ns
	Day 189 (6 months)	41 (20)	*83*	42 (24)	*27*	ns
Aspartate aminotransferase (U/L)	Basal	42 (26)	*83*	36 (11)	*27*	ns
(AST)	Day 44 (1,5 months)	43 (40)	*81*	35 (11)	*28*	ns
	Day 189 (6 months)	39 (41)	*83*	35 (10)	*27*	ns
Urea (mmol/L)	Basal	6.3 (2.5)	*81*	6.8 (3.0)	*26*	ns
	Day 44 (1,5 months)	7.8 (2.9)	*75*	7.1 (2.5)	*26*	ns
	Day 189 (6 months)	8.3 (4.2)	*65*	7.5 (6.4)	*24*	*
Creatinine (µmol/L)	Basal	97 (28)	*81*	90 (21)	*26*	ns
	Day 44 (1,5 months)	101 (42)	*75*	96 (29)	*26*	ns
	Day 189 (6 months)	109 (62)	*65*	97 (31)	*24*	ns
Total Protein (g/L)	Basal	73 (7)	*81*	73 (7)	*26*	ns
	Day 44 (1,5 months)	69 (8)	*75*	71 (8)	*26*	ns
	Day 189 (6 months)	66 (10)	*65*	67 (6)	*24*	ns
Albumin (g/L)	Basal	32 (6)	*81*	33 (5)	*26*	ns
	Day 44 (1,5 months)	31 (5)	*75*	32 (5)	*26*	ns
	Day 189 (6 months)	31 (6)	*65*	31 (5)	*24*	ns
Cholesterol (mmol/L)	Basal	5.4 (1.7)	*81*	5.2 (1.5)	*26*	ns
	Day 44 (1,5 months)	5.5 (1.5)	*75*	5.5 (1.3)	*26*	ns
	Day 189 (6 months)	5.3 (1.3)	*64*	4.9 (1.3)	*23*	ns
Glucose (mmol/L)	Basal	4.2 (1.5)	*81*	4.4 (1.4)	*26*	ns
	Day 44 (1,5 months)	4.2 (1.3)	*75*	4.5 (1.0)	*26*	ns
	Day 189 (6 months)	4.3 (1.4	*65*	4.7 (0.8)	*24*	ns
Urinary specific gravity	Basal	1.03 (0.01)	*80*	1.02 (0.01)	*25*	ns
	Day 44 (1,5 months)	1.04 (0.03)	*75*	1.03 (0.01)	*25*	ns
	Day 189 (6 months)	1.05 (0.06)	*60*	1.03 (0.01)	*23*	ns
Urinary pH	Basal	7.0 (0.8)	*81*	6.9 (0.9)	*25*	ns
	Day 44 (1,5 months)	7.0 (0.8)	*75*	7.0 (0.8)	*25*	ns
	Day 189 (6 months)	6.9 (0.8)	*60*	6.9 (0.9)	*23*	ns

ns: p>0.05, * p<0.05, ** <0.01.

Digestive tract disorders reported as AE, classified as A, B or O.

Case #	Group	Description
H-02-02	Enflicoxib	Ten years old mixed breed female. After 3 months of treatment, it shows vomiting and treated symptomatically with famotidine and recovers completely without discontinuing enflicoxib treatment.
H-04-01	Enflicoxib	Female Leonberger female of 10 years old. After seven doses of treatment the dog starts showing apathy, appetite loss and laboratory results show macrocytic hypochromic regenerative anaemia compatible with GI bleeding and FOB+. The dog was withdrawn from the study, treated with famotidine, sucralfate and catosal, and recovered completely.
H-02-14	Enflicoxib	Thirteen years old Dachshund male. After four doses of treatment the dog shows vomiting and treated symptomatically with famotidine and recovers completely without discontinuing enflicoxib treatment.
H-01-04	Enflicoxib	Nine years old male German Shepard. After 14 doses the dog owner saw blood in the faeces. The dog was withdrawn (due to also worsening of OA symptoms, the dog could not stand up) and symptomatic treatment with famotidine and sucralfate was started and the dog recovered completely after five days.
P-02-06	Enflicoxib	Eleven years old mixed breed male. After 7 doses the dog shows bloody diarrhoea. Treated with probiotics. The dog was withdrawn by owner decision but recovered completely.
P-02-08	Enflicoxib	Ten years old English Pointer male. After 6 doses of treatment the dog shows moderate diarrhoea for less than 48 hours and recovered completely without treatment. Enflicoxib treatment was not discontinued until the end of the study.
H-02-08	Placebo	Ten years old Jack Terrier male. After 3 months of treatment with placebo the dog shows diarrhoea and vomiting. Treated with probiotics, sucralfate, famotidine, and B vitamins complex and recovered completely. The dog continued in the study

Adverse Events as described in:

Salichs M, Badiella L, Sarasola P, Homedes J. Efficacy and safety of enflicoxib for treatment of canine osteoarthritis: A 6-week randomised, controlled, blind, multicentre clinical trial. Vet Rec. 2022 Sep;191(6):e949.

Summary of AEs reported classified as “A”, “B” or “O” according to the type of event.

Number of animals presenting AEs (%)	Enflicoxib 4 mg/kg n=61	Enflicoxib 2 mg/kg n=60	Placebo n=63	Mavacoxib n=58	Total n=242
Emesis or nausea	7 (11.5)	3 (5.0)	8 (12.7)	4 (6.9)	22 (9.1)
Diarrhoea or pasty stools	5 (8.2)	4 (6.7)	4 (6.3)	3 (5.2)	16 (6.6)
Apathy	2 (3.3)	1 (1.7)	0	0	3 (1.2)
Polydipsia	1 (1.6)	0	1 (1.6)	0	2 (0.8)
Weight loss	0	1 (1.7)	1 (1.6)	0	2 (0.8)
Abdominal pain	0	0	1 (1.6)	0	1 (0.4)
Constipation	0	0	0	1 (1.7)	1 (0.4)
Acute renal failure	0	0	0	1 (1.7)	1 (0.4)
Increased salivation	1 (1.6)	0	0	0	1 (0.4)
Total*	12 (19.7)	6 (10%)	9 (14.3)	6 (10.3)	33 (13.6)

*Some of the AEs reported included more than one clinical sign in the same dog

Description of the reported AEs related to the GI system.

	Enflicoxib 4 mg/kg n=61	Enflicoxib 2 mg/kg n=60	Placebo n=63	Mavacoxib n=58
Emesis and nausea				
Number of events	9	3	8	6
Mean duration (days)	1.4	3	1.6	4.2
Duration range (days)	1-3	1-6*	1-5*	1-14*
Treatment needed#	2	0	2	2
Diarrhoea / pasty stools				
Number of events	7	3	6	3
Mean duration (days)	3	5.6	6.6	2
Duration range (days)	1-4	2-8	2-18*	1-4
Treatment needed#	3	1	3	0

*one animal

treatments included probiotics, antiemetics, antacids, gastric protectants, nutritional supplements, and antibiotics.

Digestive tract disorders reported as AE, classified as A, B or O as described in:

Salichs M, Badiella L, Sarasola P, Homedes J. Enflicoxib for canine osteoarthritis: A randomized, blind, multicentre, non-inferiority clinical trial compared to mavacoxib. PLoS One. 2022 Sep 20;17(9):e0274800.

Enflicoxib Case #	Description	Mavacoxib Case #	Description
BAZ01	Vomiting once the day after first product administration. Not treated	BUS05	Diarrhoea for one week after second product administration. Treated with antimicrobial and probiotic
BUS04	Vomited in the car on its way back home, 1-1.5 hours after first product administration. Not treated	CAR09	Emesis 15-20 minutes after second product administration. Not treated
BUS07	Bilious emesis. Three episodes after third product administration. Treated with antiemetics	DER02	Emesis once the day after first product administration, and diarrhoea for two days after second product administration. Not treated
GON03	Vomited in the car on its way back home, 1 hour after second product administration. Not treated	FER03	Diarrhoea for four days after second product administration. Not treated
MAS07	Emesis and dehydration two days before diagnosing a perforated gastric ulcer after fourth product administrations. Treated with fluids, antimicrobial, antispasmodic and antacid.	NAV07	Emesis for two days between administrations. Treated with antiulcer.
PRA08	Several episodes of emesis between second and third product administration. Associated with diarrhoea and apathy during 24hours. Not treated.	ROD04	Single vomit and loss of appetite the day of second product administration. Not treated
RUI02	Emesis with food content, once between first and second product administration. Not treated	ROD05	Soft faeces for several days at the end of the study. Not treated
ROD06	Soft faeces for two days at the end of the study. Not treated	RUI01	Diarrhoea for four days after first product administration. Not treated
SAN01	Emesis once the day of fourth product administration. Regurgitation reported on a previous day. Not treated	RUI14	Diarrhoea for two days several days after second product administration. Not treated
SAN10	Emesis once the day of fourth product administration. Loss of appetite previously reported. Not treated	RUI18	Emesis for several days after first product administration associated with haemorrhagic diarrhoea, hypothermia (36.1ºc) depression and alteration of blood and renal parameters. Treated with antimicrobial, fluids, antiulcer and antiemetic
SAN17	Emesis once the day before second product administration. Not treated		

AE: Adverse Event; A=probable, B=possible, O=unclassifiable/unassessable

Figure S1. Relationship between selected covariates and plasma levels of enflicoxib and the pyrazol metabolite on day 44 and 189.

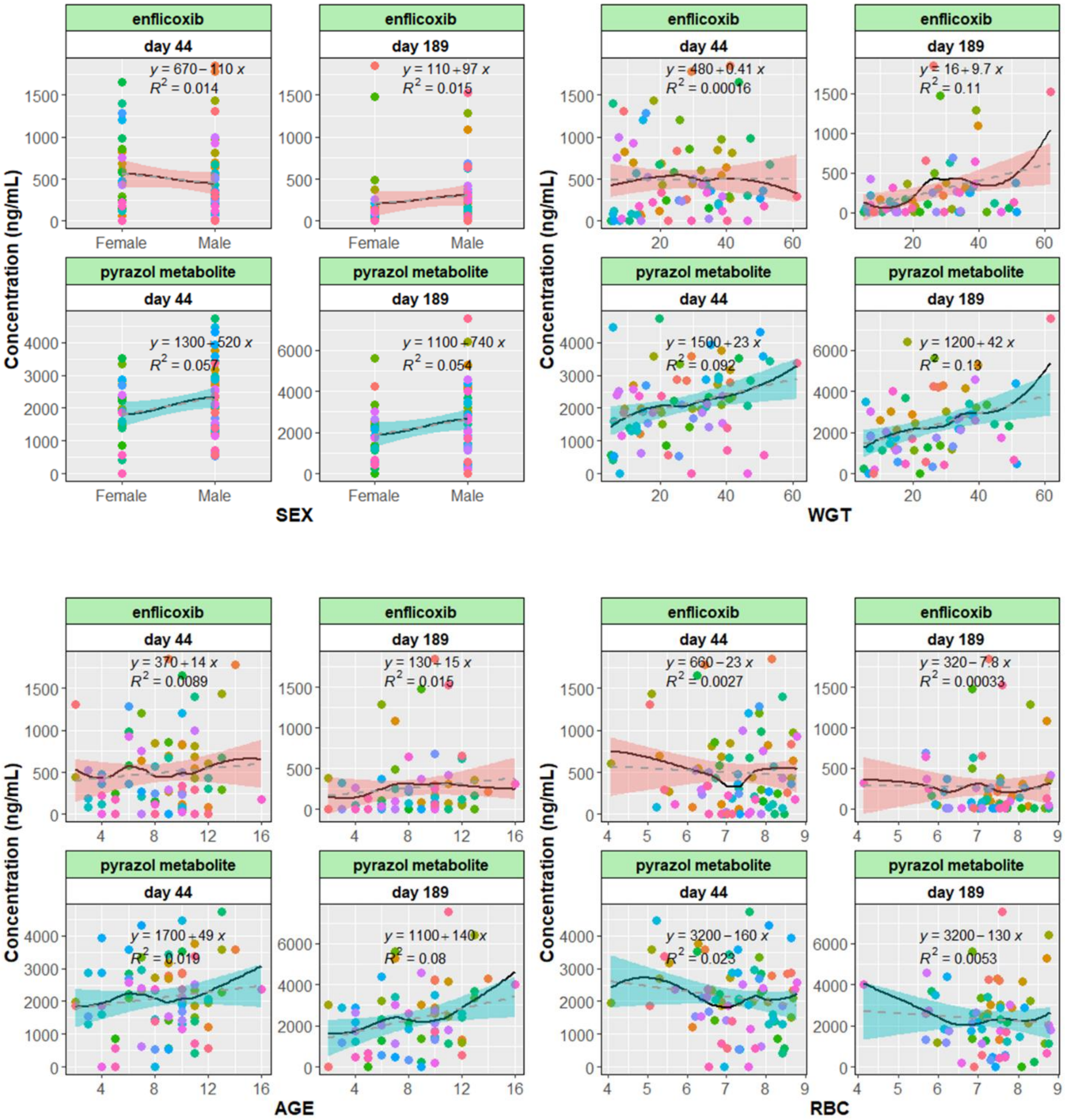

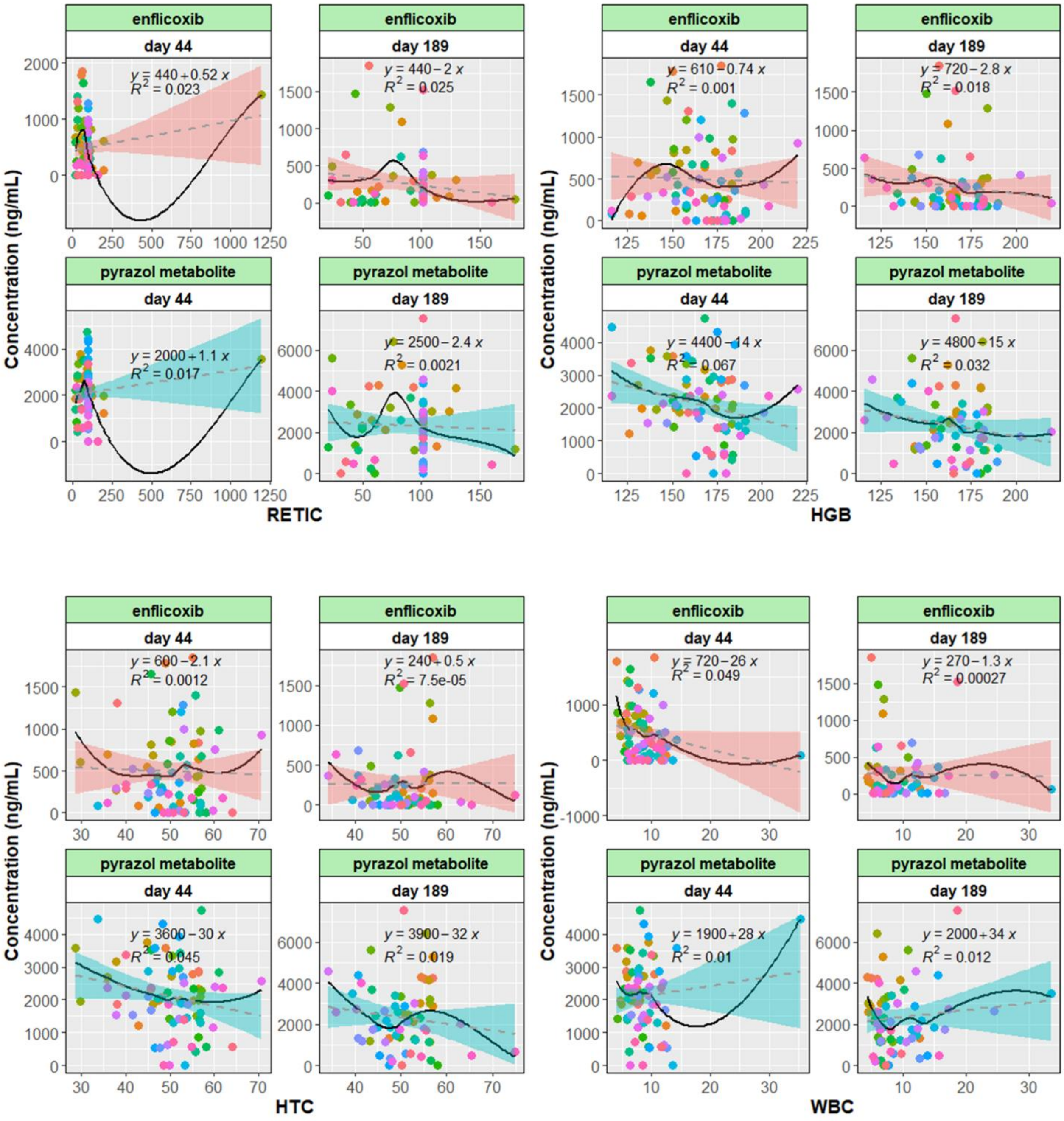
enflicoxib
day 44
y = 440 + 0.52 x
R^2 = 0.023
day 189
y = 440 − 2 x
R^2 = 0.025
pyrazol metabolite
y = 2000 + 1.1 x
R^2 = 0.017
y = 2500 − 2.4 x
R^2 = 0.0021
RETIC
y = 610 − 0.74 x
R^2 = 0.001
y = 720 − 2.8 x
R^2 = 0.018
y = 4400 − 14 x
R^2 = 0.067
y = 4800 − 15 x
R^2 = 0.032
HGB
y = 600 − 2.1 x
R^2 = 0.0012
y = 240 + 0.5 x
R^2 = 7.5e-05
y = 3600 − 30 x
R^2 = 0.045
y = 3900 − 32 x
R^2 = 0.019
HTC
y = 720 − 26 x
R^2 = 0.049
y = 270 − 1.3 x
R^2 = 0.00027
y = 1900 + 28 x
R^2 = 0.01
y = 2000 + 34 x
R^2 = 0.012
WBC
Concentration (ng/mL)

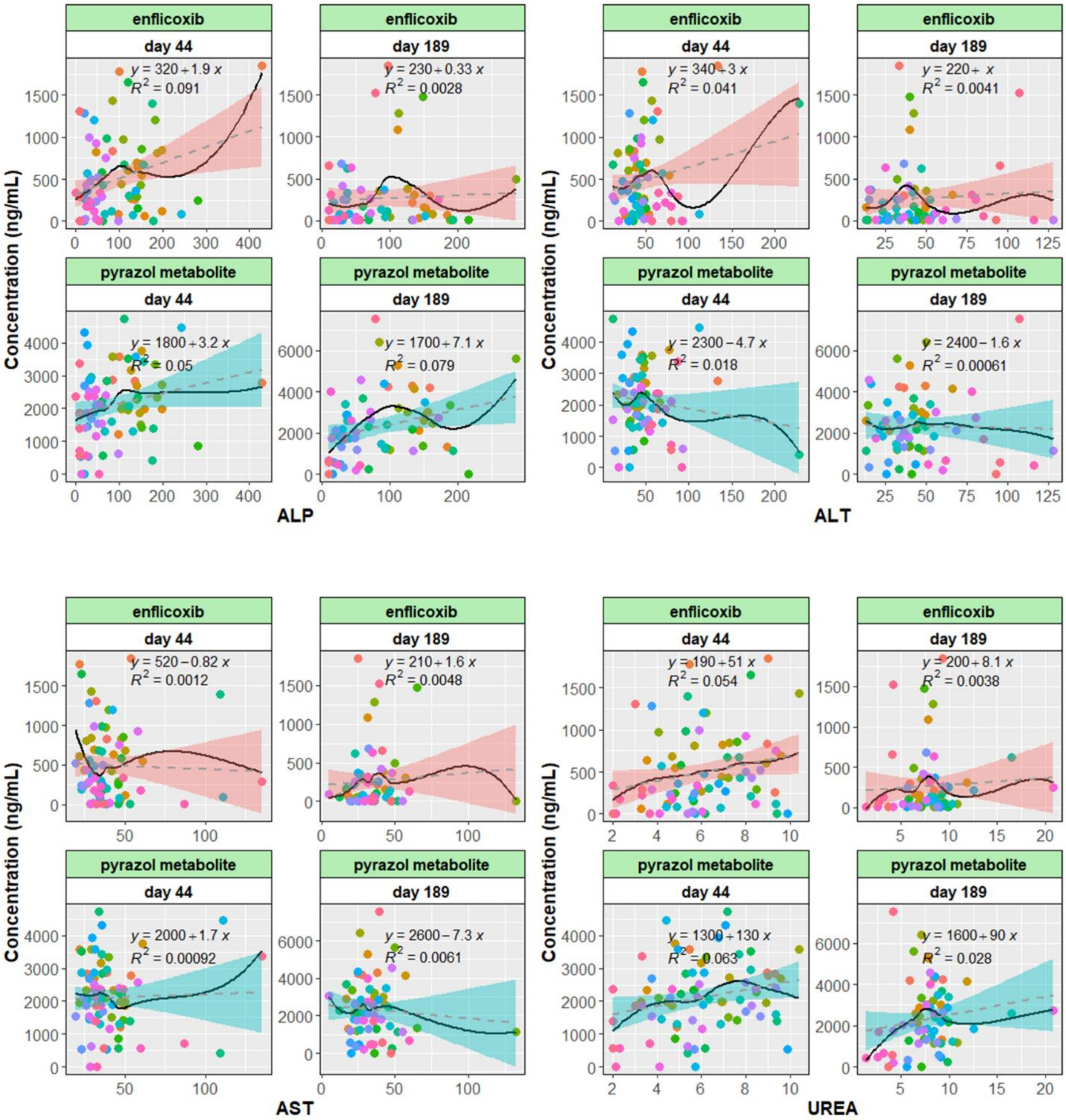
enflicoxib
day 44
y = 320 + 1.9 x
R^2 = 0.091
day 189
y = 230 + 0.33 x
R^2 = 0.0028
pyrazol metabolite
y = 1800 + 3.2 x
R^2 = 0.05
y = 1700 + 7.1 x
R^2 = 0.079
ALP
y = 340 + 3 x
R^2 = 0.041
y = 220 + x
R^2 = 0.0041
y = 2300 − 4.7 x
R^2 = 0.018
y = 2400 − 1.6 x
R^2 = 0.00061
ALT
y = 520 − 0.82 x
R^2 = 0.0012
y = 210 + 1.6 x
R^2 = 0.0048
y = 2000 + 1.7 x
R^2 = 0.00092
y = 2600 − 7.3 x
R^2 = 0.0061
AST
y = 190 + 51 x
R^2 = 0.054
y = 200 + 8.1 x
R^2 = 0.0038
y = 1300 + 130 x
R^2 = 0.063
y = 1600 + 90 x
R^2 = 0.028
UREA
Concentration (ng/mL)

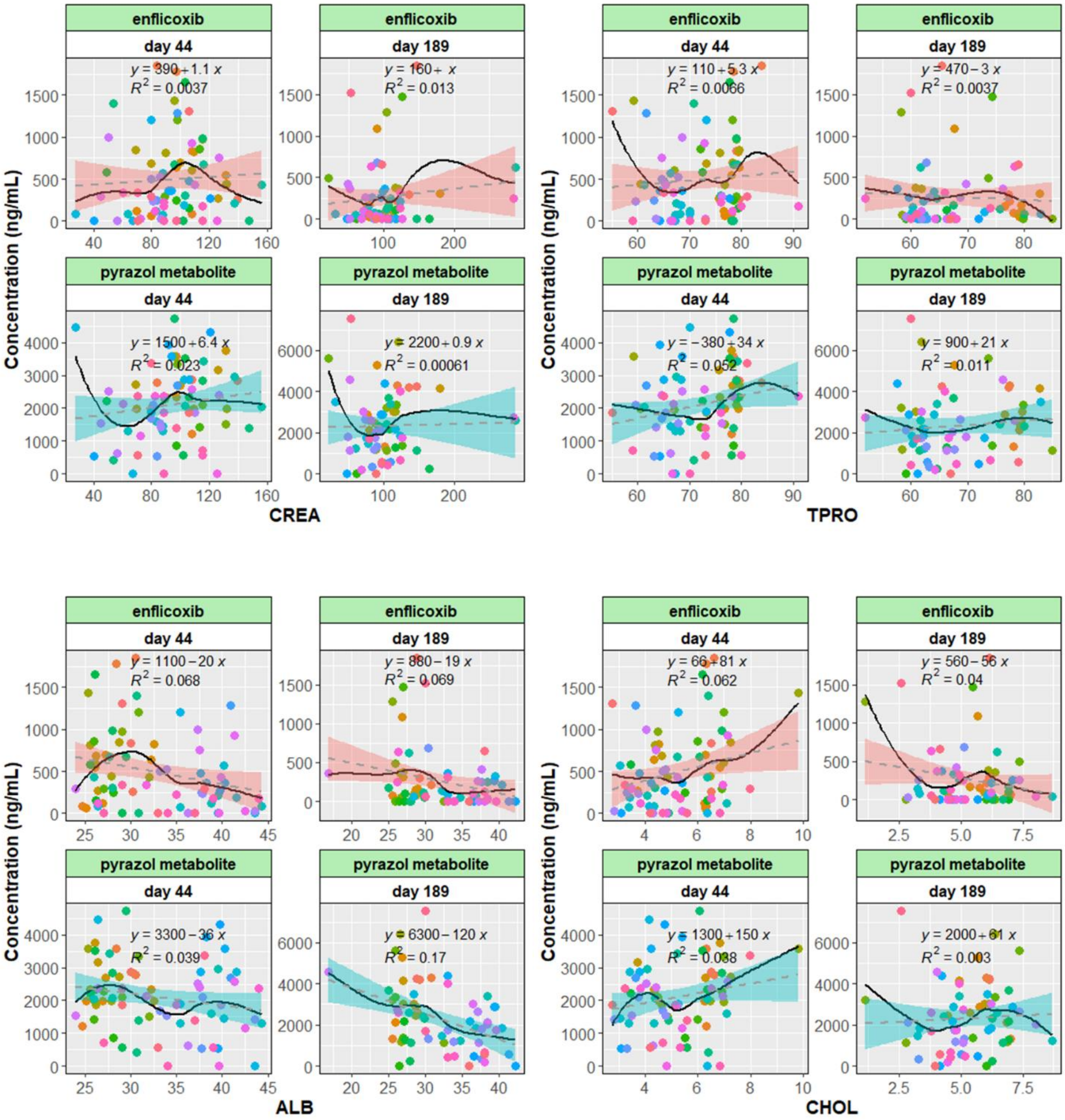
enflicoxib
day 44
day 189
pyrazol metabolite
y = 390 + 1.1 x
R^2 = 0.0037
y = 160 + x
R^2 = 0.013
y = 1500 + 6.4 x
R^2 = 0.023
y = 2200 + 0.9 x
R^2 = 0.00061
y = 110 + 5.3 x
R^2 = 0.0066
y = 470 − 3 x
R^2 = 0.0037
y = −380 + 34 x
R^2 = 0.052
y = 900 + 21 x
R^2 = 0.011
y = 1100 − 20 x
R^2 = 0.068
y = 880 − 19 x
R^2 = 0.069
y = 3300 − 36 x
R^2 = 0.039
y = 6300 − 120 x
R^2 = 0.17
y = 66 + 81 x
R^2 = 0.062
y = 560 − 56 x
R^2 = 0.04
y = 1300 + 150 x
R^2 = 0.038
y = 2000 + 61 x
R^2 = 0.003
Concentration (ng/mL)
CREA
TPRO
ALB
CHOL

Figure S2. Individual predicted PK profiles of enflicoxib and the pyrazol metabolite determined by Bayesian approach in all dogs treated with enflicoxib.

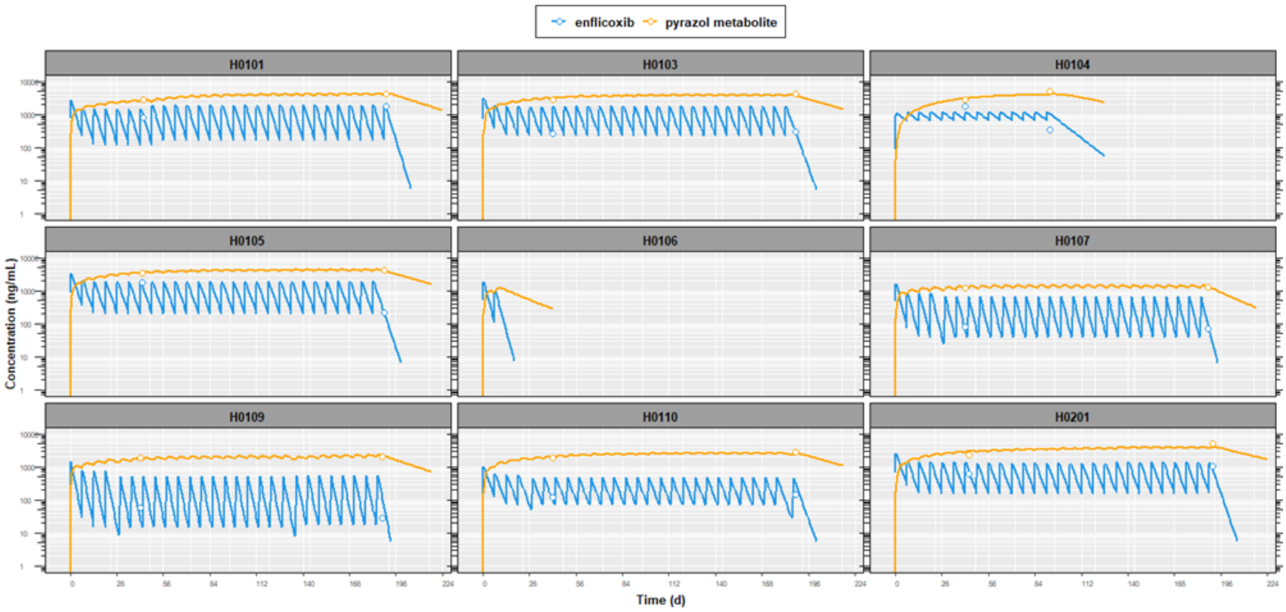

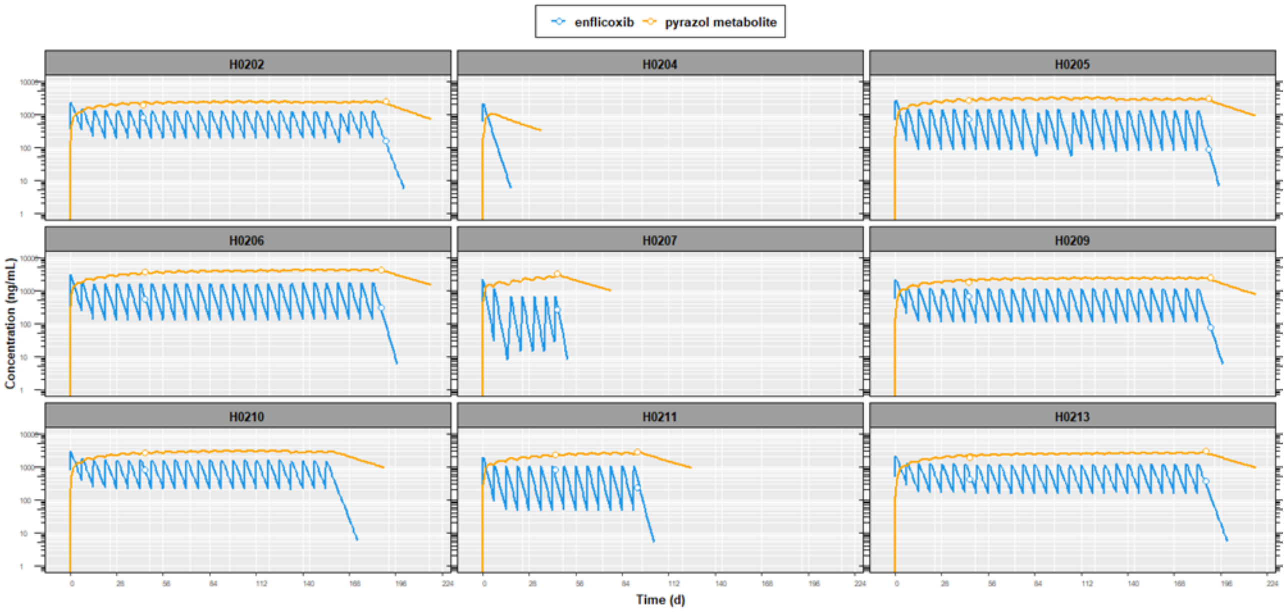

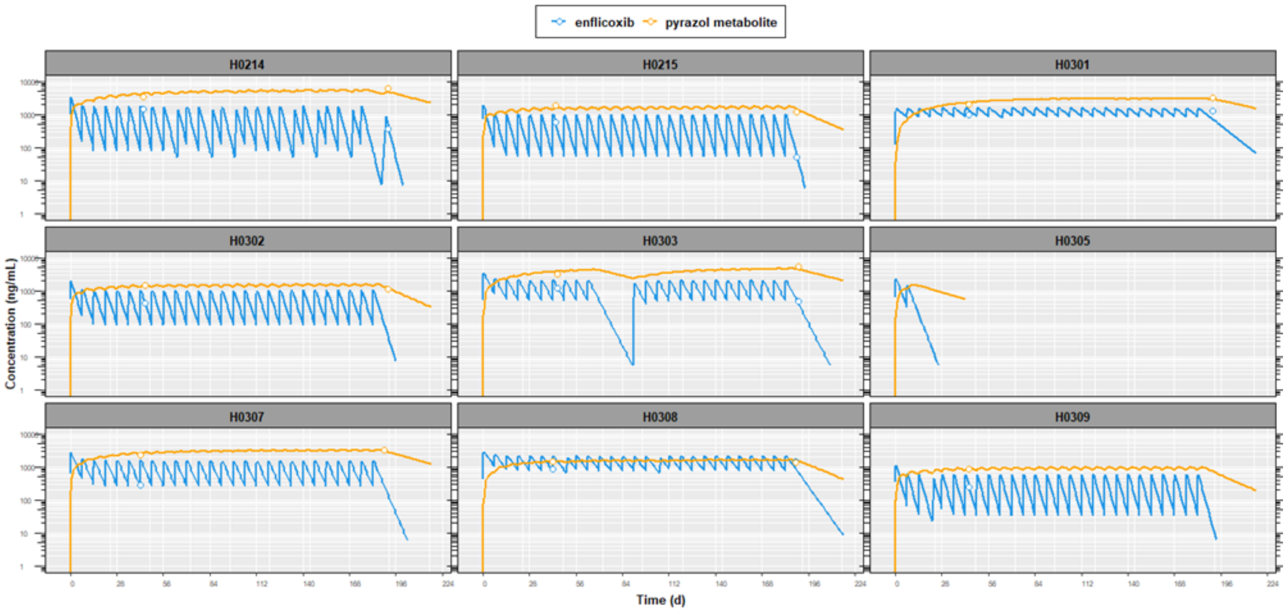
enflicoxib
pyrazol metabolite
H0214
H0215
H0301
H0302
H0303
H0305
H0307
H0308
H0309
Concentration (ng/mL)
Time (d)

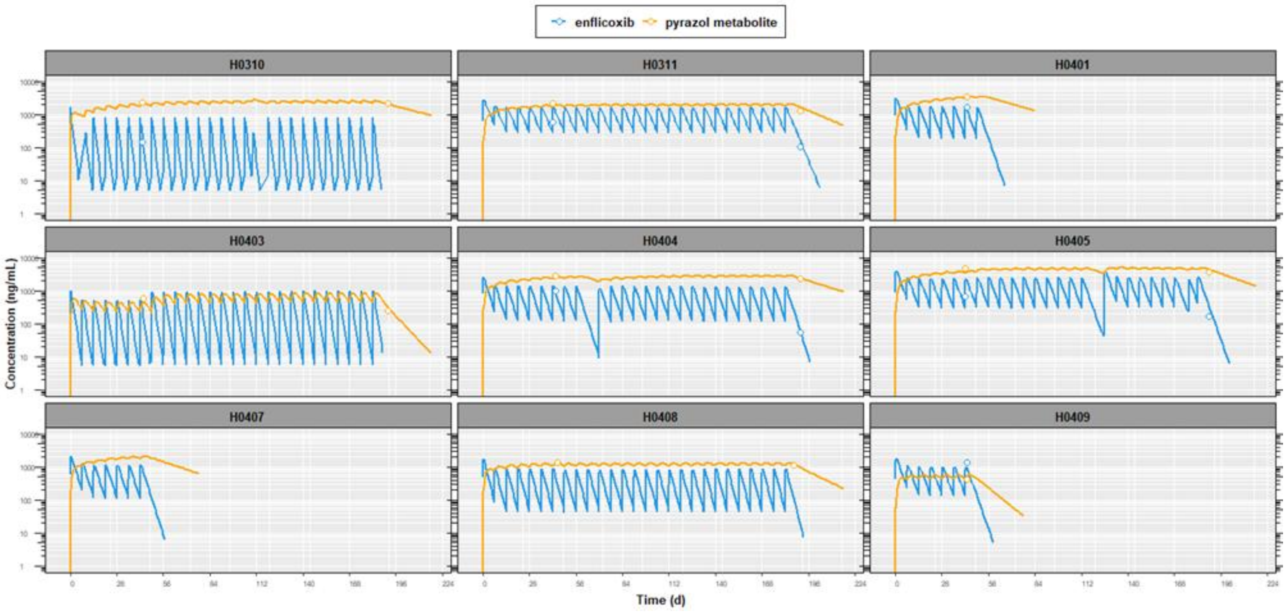
enflicoxib
pyrazol metabolite
H0310
H0311
H0401
H0403
H0404
H0405
H0407
H0408
H0409
Concentration (ng/mL)
Time (d)

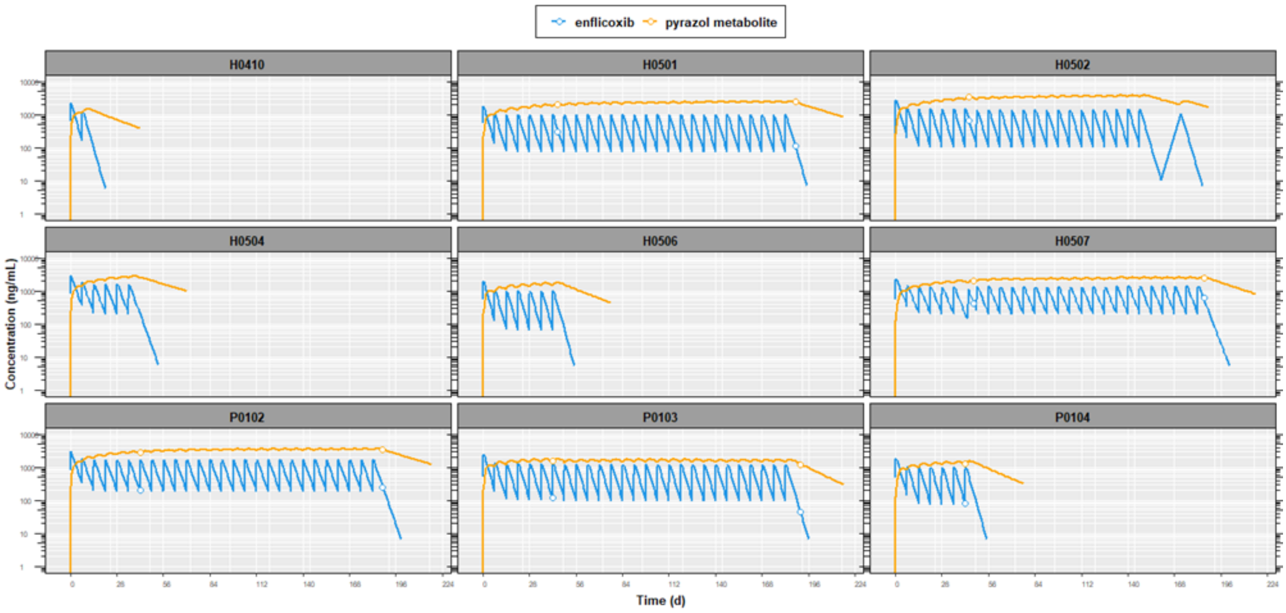
enflicoxib
pyrazol metabolite
H0410
H0501
H0502
H0504
H0506
H0507
P0102
P0103
P0104
Concentration (ng/mL)
Time (d)

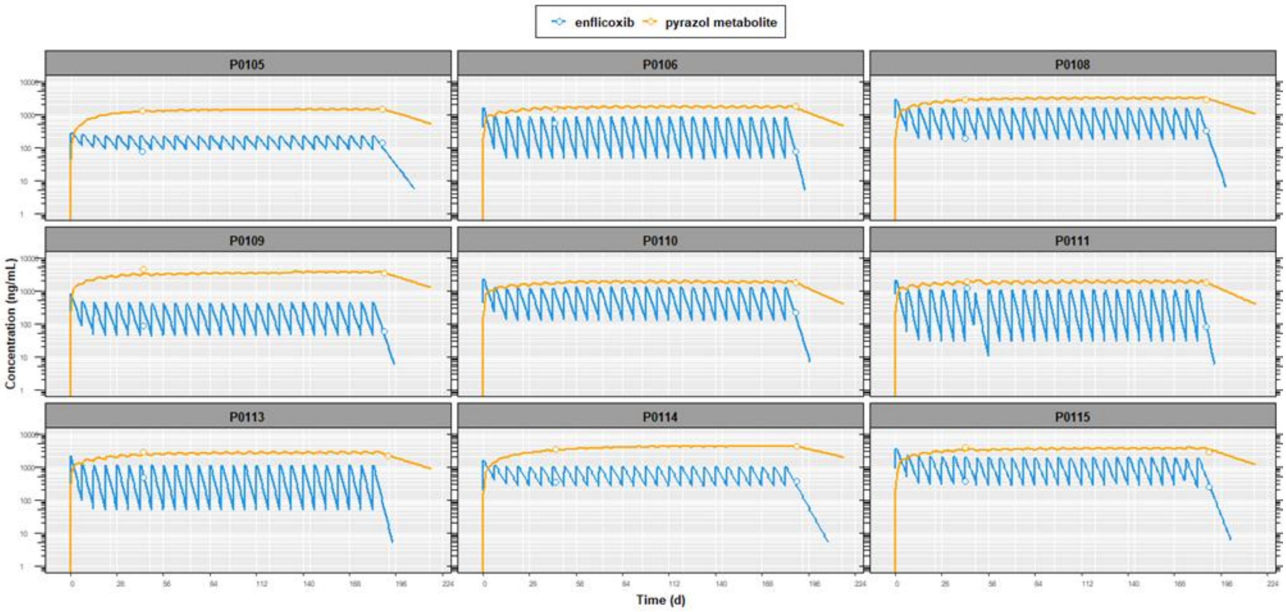
enflicoxib
pyrazol metabolite
P0105
P0106
P0108
P0109
P0110
P0111
P0113
P0114
P0115
Concentration (ng/mL)
Time (d)

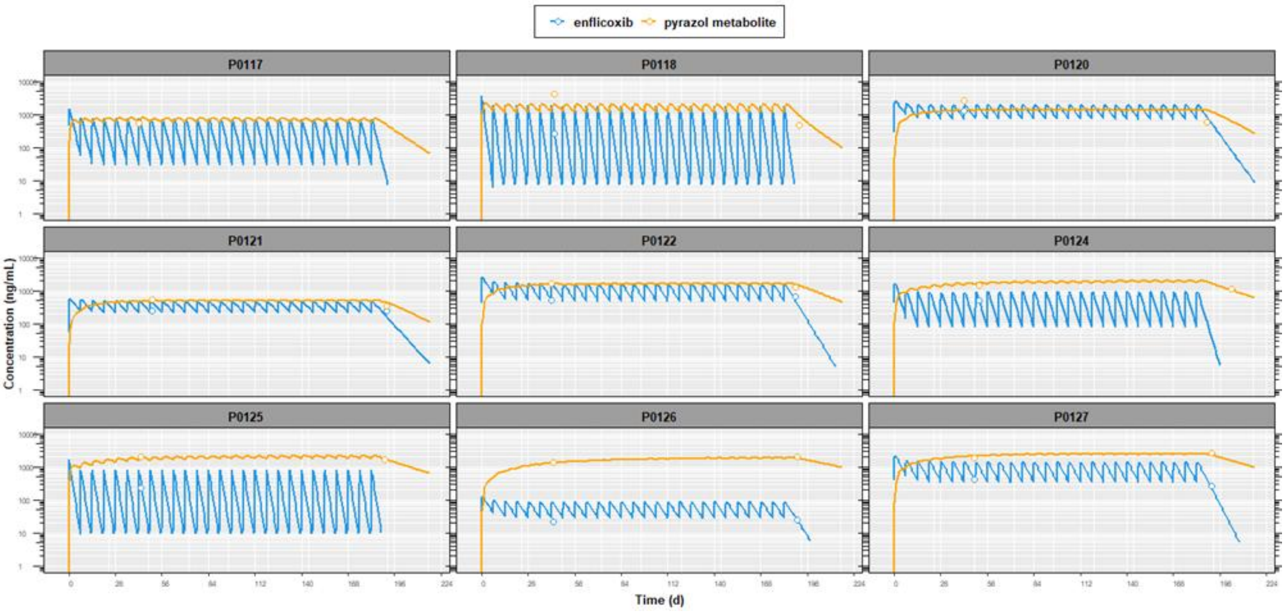
enflicoxib
pyrazol metabolite
P0117
P0118
P0120
P0121
P0122
P0124
P0125
P0126
P0127
Concentration (ng/mL)
Time (d)

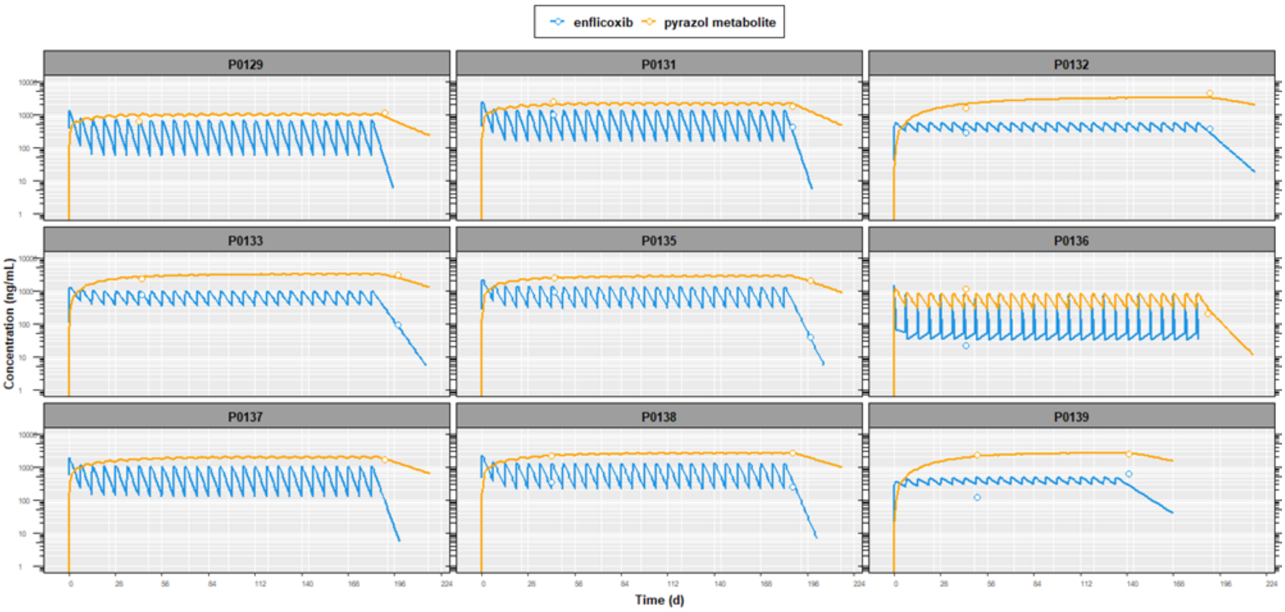
enflicoxib
pyrazol metabolite
P0129
P0131
P0132
P0133
P0135
P0136
P0137
P0138
P0139
Concentration (ng/mL)
Time (d)

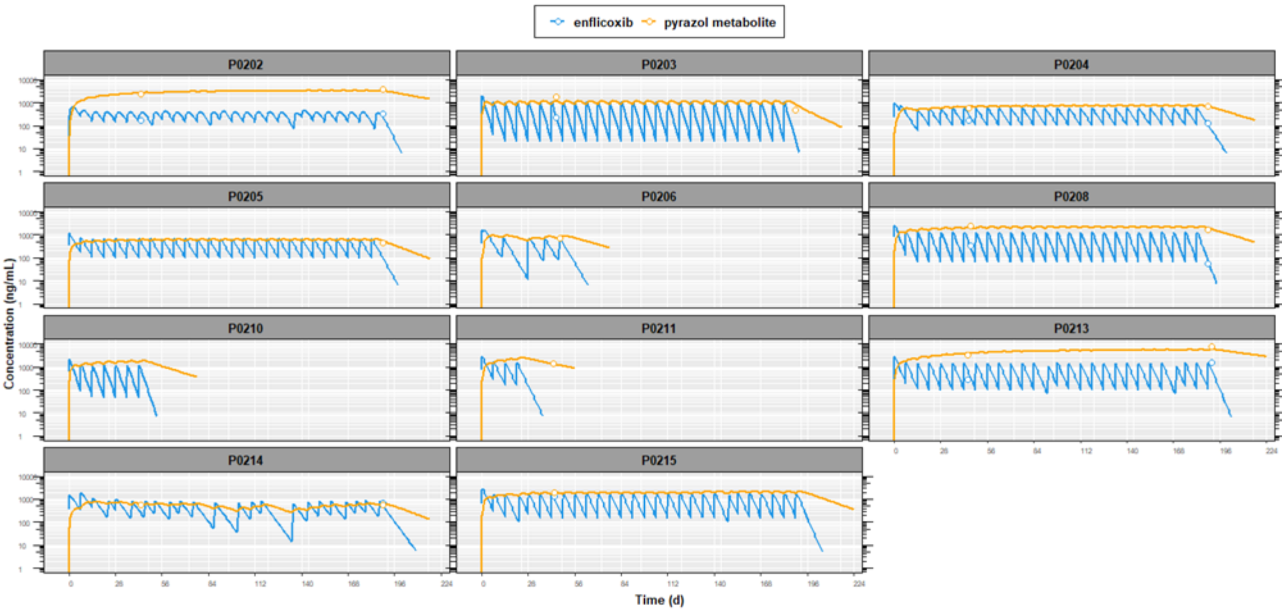

enflicoxib
pyrazol metabolite
P0202
P0203
P0204
P0205
P0206
P0208
P0210
P0211
P0213
P0214
P0215
Concentration (ng/mL)
Time (d)

Figure S3. Distribution of main PK parameters C_{max} (ng/mL), C_{min} (ng/mL), AUC_{τ} (ng/mL·d) and $T_{1/2}$ (d) (mean ±SD) for enflicoxib on weeks 4, 8, 12, 16, 20, 24 and 26.

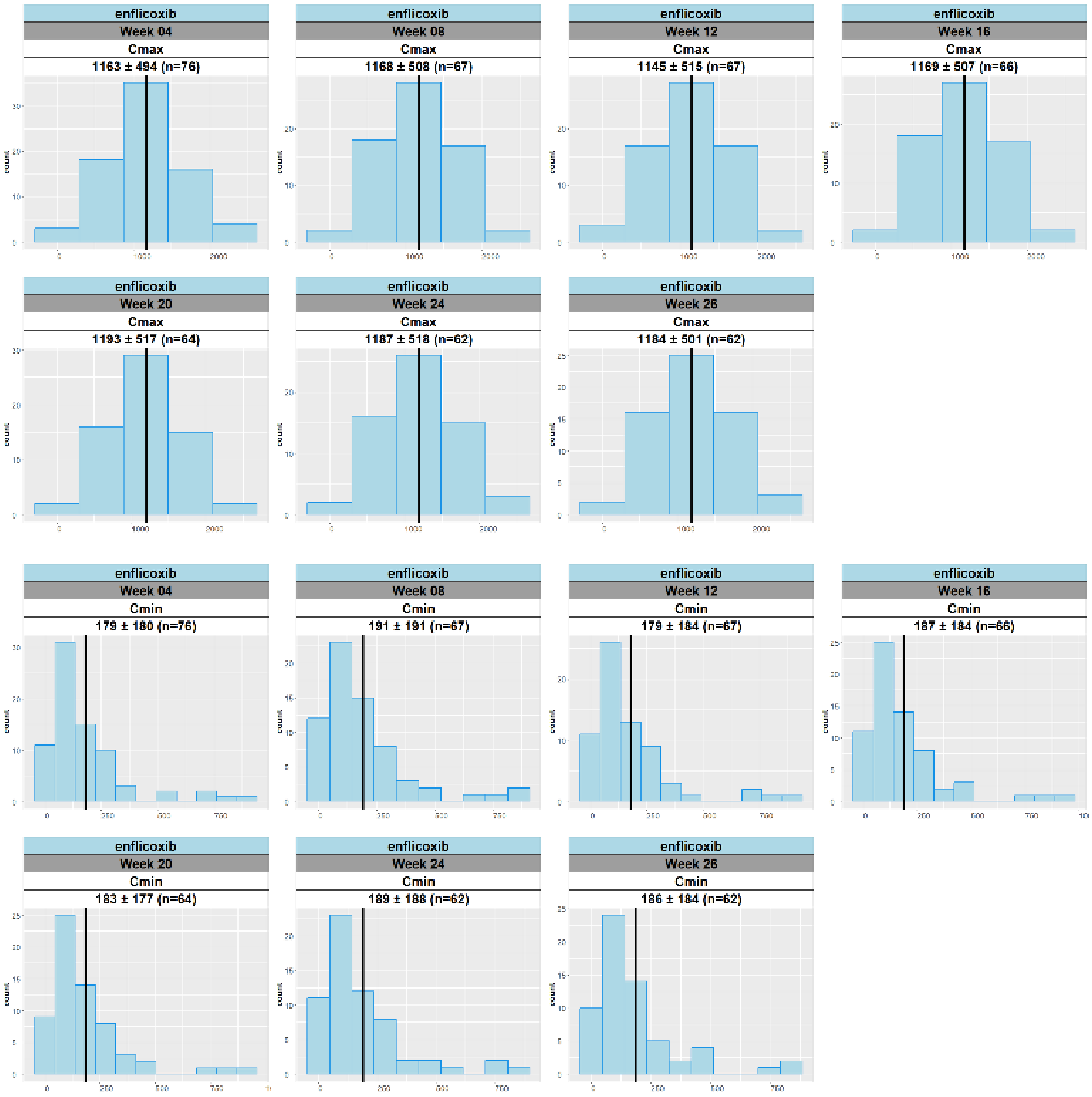

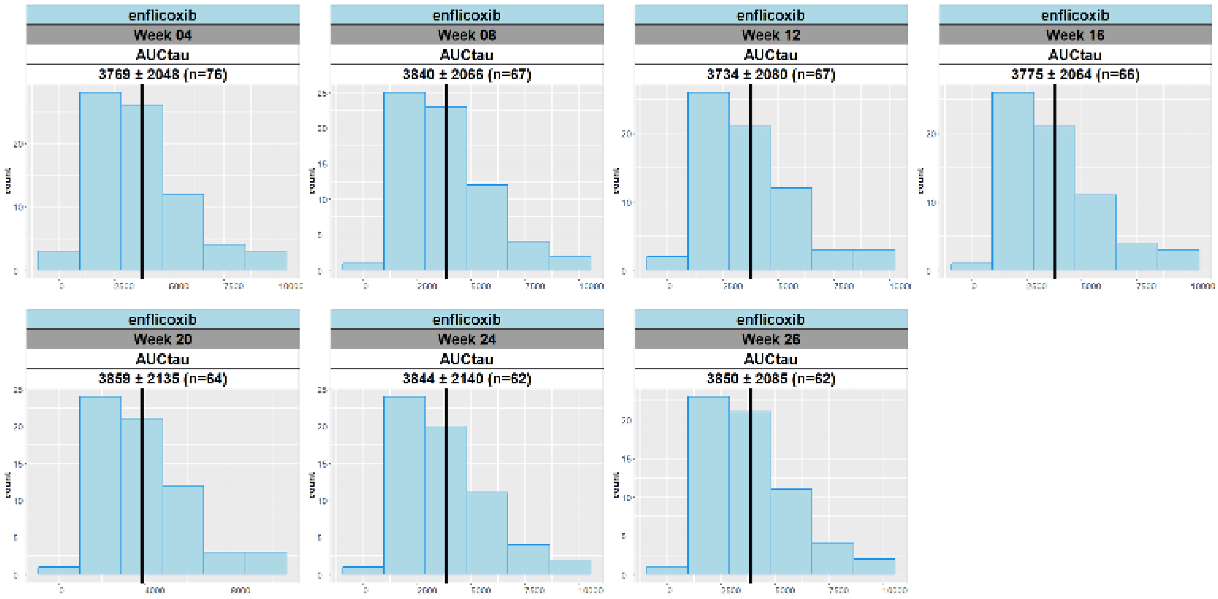
enflicoxib
Week 04
AUCtau
3769 ± 2048 (n=76)
enflicoxib
Week 08
AUCtau
3840 ± 2066 (n=67)
enflicoxib
Week 12
AUCtau
3734 ± 2080 (n=67)
enflicoxib
Week 16
AUCtau
3775 ± 2064 (n=66)
enflicoxib
Week 20
AUCtau
3859 ± 2135 (n=64)
enflicoxib
Week 24
AUCtau
3844 ± 2140 (n=62)
enflicoxib
Week 26
AUCtau
3850 ± 2085 (n=62)

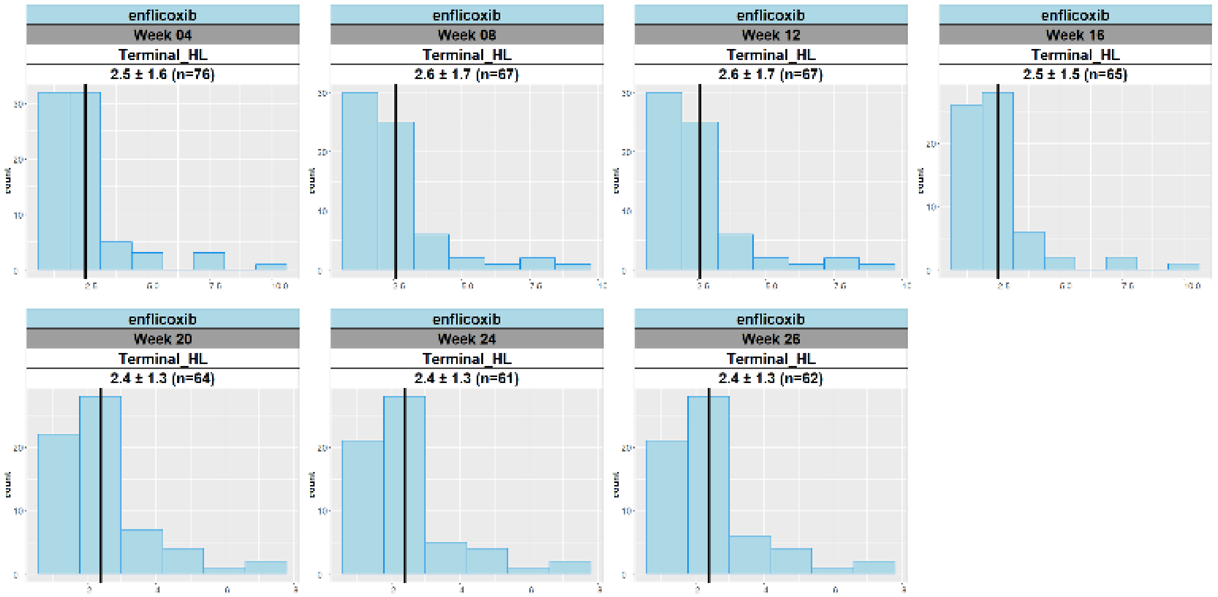
enflicoxib
Week 04
Terminal_HL
2.5 ± 1.6 (n=76)
enflicoxib
Week 08
Terminal_HL
2.6 ± 1.7 (n=67)
enflicoxib
Week 12
Terminal_HL
2.6 ± 1.7 (n=67)
enflicoxib
Week 16
Terminal_HL
2.5 ± 1.5 (n=65)
enflicoxib
Week 20
Terminal_HL
2.4 ± 1.3 (n=64)
enflicoxib
Week 24
Terminal_HL
2.4 ± 1.3 (n=61)
enflicoxib
Week 26
Terminal_HL
2.4 ± 1.3 (n=62)

Figure S4. Distribution of main PK parameters C_{max} (ng/mL), C_{min} (ng/mL), AUC_{τ} (ng/mL·d) and $T_{1/2}$ (d) (mean ±SD) for pyrazol metabolite on weeks 4, 8, 12, 16, 20, 24 and 26.

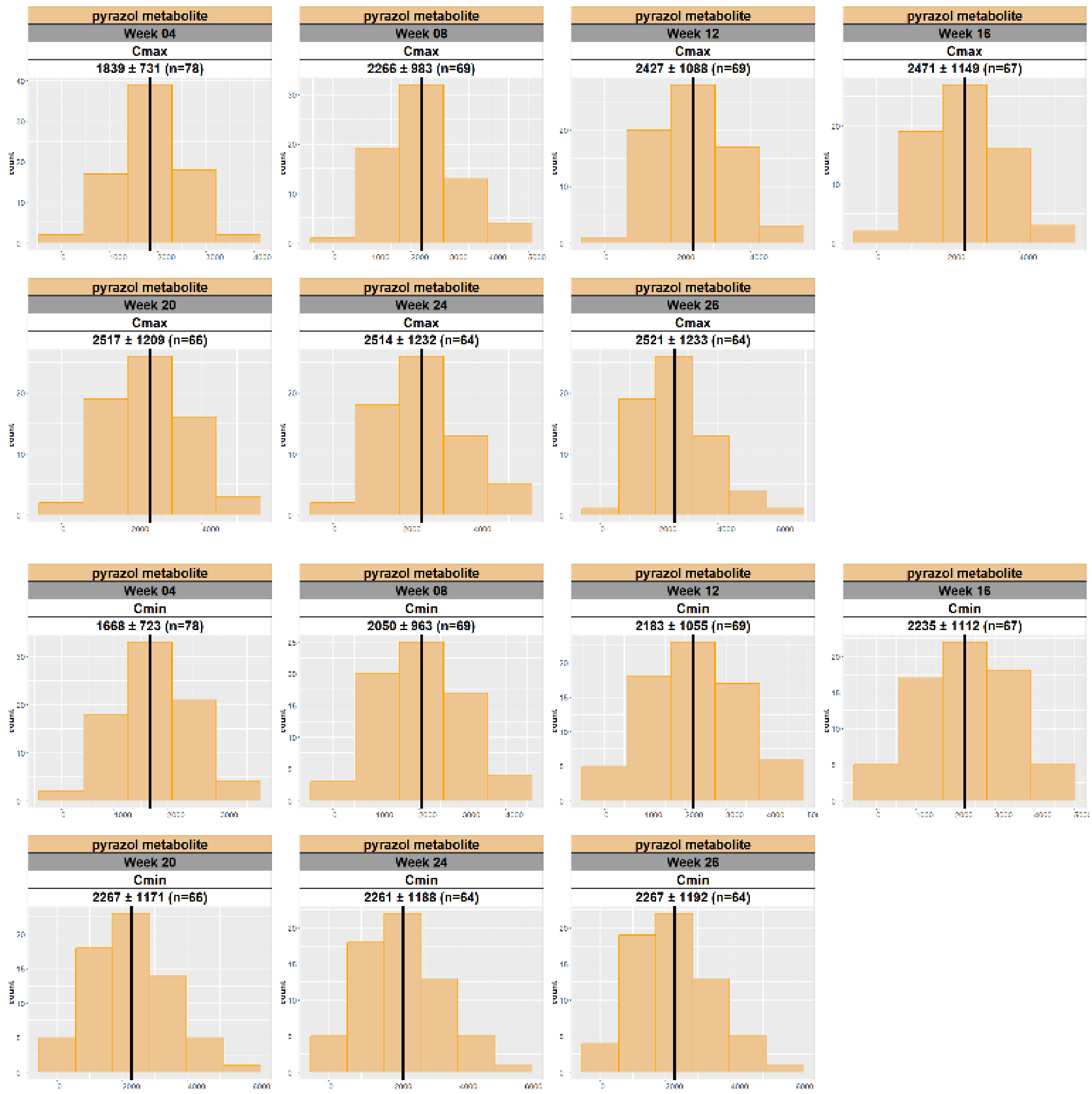

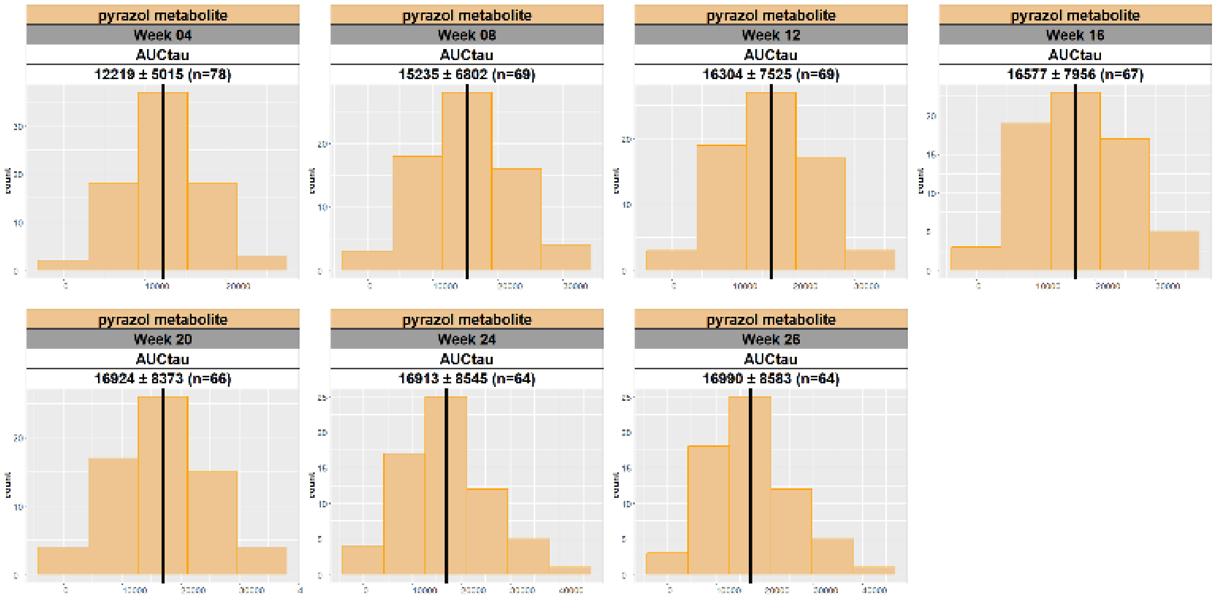
pyrazol metabolite
Week 04
AUCtau
12219 ± 5015 (n=78)
pyrazol metabolite
Week 08
AUCtau
15235 ± 6802 (n=69)
pyrazol metabolite
Week 12
AUCtau
16304 ± 7525 (n=69)
pyrazol metabolite
Week 16
AUCtau
16577 ± 7956 (n=67)
pyrazol metabolite
Week 20
AUCtau
16924 ± 8373 (n=66)
pyrazol metabolite
Week 24
AUCtau
16913 ± 8545 (n=64)
pyrazol metabolite
Week 26
AUCtau
16990 ± 8583 (n=64)
count

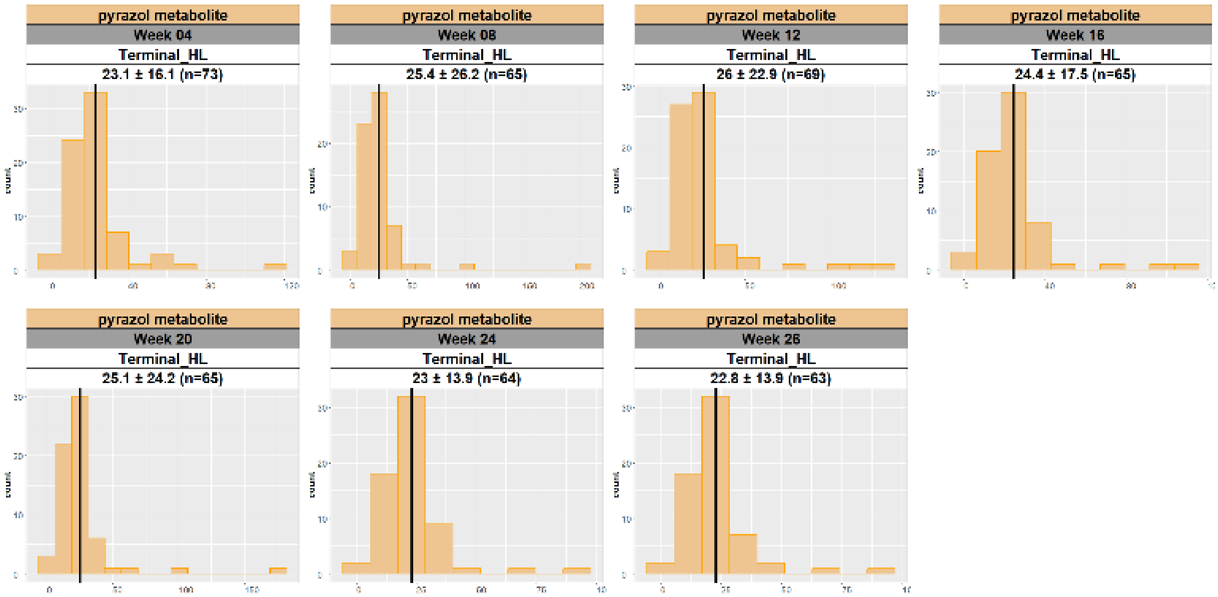
pyrazol metabolite
Week 04
Terminal_HL
23.1 ± 16.1 (n=73)
pyrazol metabolite
Week 08
Terminal_HL
25.4 ± 26.2 (n=65)
pyrazol metabolite
Week 12
Terminal_HL
26 ± 22.9 (n=69)
pyrazol metabolite
Week 16
Terminal_HL
24.4 ± 17.5 (n=65)
pyrazol metabolite
Week 20
Terminal_HL
25.1 ± 24.2 (n=65)
pyrazol metabolite
Week 24
Terminal_HL
23 ± 13.9 (n=64)
pyrazol metabolite
Week 26
Terminal_HL
22.8 ± 13.9 (n=63)
count
